# Evolutionary Complexity of Primate Immune System Uncovered by the Extensive Phylogenomic Sampling

**DOI:** 10.1093/gbe/evag087

**Published:** 2026-04-01

**Authors:** Xiuping Zhang, Boyang Wu, Yong Shao

**Affiliations:** State Key Laboratory of Genetic Evolution & Animal Models, Kunming Institute of Zoology, Chinese Academy of Sciences, Kunming 650201, China; State Key Laboratory of Genetic Evolution & Animal Models, Kunming Institute of Zoology, Chinese Academy of Sciences, Kunming 650201, China; Kunming College of Life Science, University of Chinese Academy of Sciences, Kunming, Yunnan, China; State Key Laboratory of Genetic Evolution & Animal Models, Kunming Institute of Zoology, Chinese Academy of Sciences, Kunming 650201, China; Kunming National High-Level Biosafety Research Center for Non-Human Primates, Kunming Institute of Zoology, Chinese Academy of Sciences, Kunming 650107, China

**Keywords:** primates, immune system complexity, evolutionary history, module, social system, disease model

## Abstract

The immune system mediates the complex interaction between pathogenic microorganisms and their hosts. Despite its significance, the evolutionary mechanisms underlying immune system complexity in primates remain largely elusive. In this study, we investigated the evolution of the primate immune system by generating the first comprehensive catalog of immune-related genes through extensive phylogenomic sampling. Our analyses uncovered substantial genetic diversity in the evolution of the primate immune system, in the form of modules that vary in their sequences and functional capabilities. We identified a novel module (Type 3c) that has experienced long-term coevolution between primates and lentiviruses over a long evolutionary timescale. Furthermore, we found that social system complexity, rather than diet or group size, may potentially shape the immune system evolution of primates. We further uncovered the evolutionary histories of key immune-associated genes, including *IFNAR2* and *C5AR1*, which are implicated in SARS-CoV-2 infection. More importantly, we revealed a divergence in the selective pressures on immune-associated genes between experimental primates and humans. This finding provides a critical caveat, suggesting that extreme caution is warranted when using these primates as models for human diseases such as HIV-1, Hepatitis C, and Influenza A. In summary, this work uncovers key evolutionary mechanisms that have fundamentally shaped the complexity of immune systems across primates.

Significance Our investigation, employing a newly developed algorithm and broad phylogenomic sampling of primates, delineates the evolutionary landscape of their immune systems. We thereby report a novel module with signatures of primate-lentivirus coevolution and demonstrate that social complexity is a more significant evolutionary driver than diet or group size. Crucially, we document variations in selective pressures on immune-associated genes between humans and model primates—a finding that urges caution in translational research employing these disease models.

## Introduction

The evolutionary dynamics of host–pathogen interactions impose persistent selective pressure on the host species (Barreiro and Quintana-Murci 2010). Consequently, this has driven the evolution of sophisticated immune systems in host species. The accelerated evolution of immune tissues relative to non-immune tissues in primates demonstrates the strong selective pressure acting on the immune system of primates (Shao et al. 2023). Owing to their close evolutionary relationship and numerous physiological parallels with humans, nonhuman primates (NHPs) are thus indispensable models for research into human evolution and diseases (Bakken et al. 2016; Estes et al. 2018; Luo et al. 2021). The immune system is central to both disease progression and the host's ability to clear pathogenic infections. Therefore, elucidating the evolutionary mechanisms of the immune system, particularly within primates, is essential for understanding human disease pathogenesis and advancing therapeutic development. Yet the evolutionary pathways that have shaped immune systems across the primate phylogeny are poorly characterized.

Differences in susceptibility and pathogenicity among primates reflect the underlying evolutionary complexity of their immune systems (Barreiro et al. 2010). Previous studies indicate that the rapid evolution of immune-associated genes underpins, at least in part, the molecular mechanisms driving immunological heterogeneity among primate species (van der Lee et al. 2017). Analyses indicate that genes involved in pathogen recognition and innate immune mediation have exhibited signatures of positive selection during primate evolution (Nakajima et al. 2008; Wlasiuk et al. 2009; Wlasiuk and Nachman 2010a). A comparative analysis revealed that immunoglobulin superfamily genes involved in immune regulation showed a pronounced acceleration in evolutionary rate (d*_N_*/d*_S_*) relative to genes assigned to other Gene Ontology terms (Ohtani et al. 2011). These evolutionary factors may underlie the observed differences in disease susceptibility. For example, while HIV-1 infects both humans and pig-tailed macaques, viral replication and pathogenicity differ markedly between the two species (Pang et al. 2023). Differential susceptibility to SARS-CoV-2 has been revealed between Old World and New World primates despite their close genetic relatedness (Lu et al. 2020; Singh et al. 2021). Currently, evolutionary pressure estimates for immune-associated genes from broad phylogenetic sampling of primates are still scarce, yet they are crucial for unraveling the evolutionary trajectory of the primate immune system.

By integrating diverse research areas such as speciation, social complexity, and genomic diversity, the largest primate genome study to date has yielded a more profound understanding of primate biology (Kuderna et al. 2023; Qi et al. 2023; Rivas-González et al. 2023; Shao et al. 2023; Wu et al. 2023; Zhang et al. 2023). The increasing availability of long-read sequenced primate genomes enables the assembly and annotation of higher-quality coding regions. These data now allow the construction of a comprehensive catalog of immune-associated orthologous genes across primates. Therefore, this study integrated immune-associated phenotypic traits with extensive high-quality genomic data across a primate phylogenetic framework. We aim to reconstruct the evolutionary history of the immune system and thereby reveal the molecular bases and evolutionary features of its diversification among primate lineages.

## Results

### Identification of Immune-Associated Datasets Under a Primate Phylogeny With the Extensive High-quality Genomic Data

**Fig. 1.**
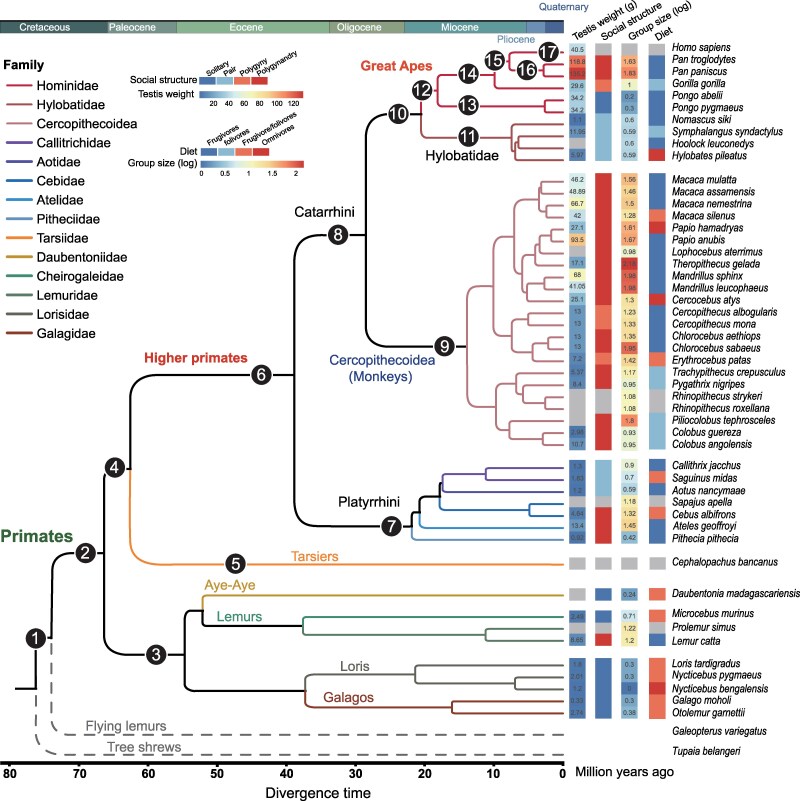
A genomic phylogeny of primates and associated immune indices. The phylogeny was from a previous study (Shao et al. 2023). The testis weight data were summarized from studies (Hill and Kanagasuntheram 1959; Harcourt et al. 1981; Harcourt et al. 1995; Kappeler 1997; Dixson and Anderson 2004; Lemaître et al. 2009; Lüpold et al. 2019). The grey box in testis weight and group size represented that the data in this species was unavailable. The social structure data were from a previous study (DeCasien et al. 2017). The grey box in social structure and diet represented that the data of this species was from a different study instead of DeCasien et al. (2017), which was excluded in our downstream analysis to ensure the accuracy of our analyses. 17 branches encoded using numerical symbols were utilized as downstream module analyses. The 17 targeted branches in this study were as follows: ① Primatomorpha ancestor, ② Primates ancestor, ③ Strepsirrhini ancestor, ④ Haplorrhini ancestor, ⑤ Tarsiiformes ancestor, ⑥ Simiiformes ancestor, ⑦ Platyrrhini ancestor, ⑧ Catarrhini ancestor, ⑨ Cercopithecidae ancestor, ⑩ Hominoidea ancestor, ⑪ Hylobatidae ancestor, ⑫ Hominidae ancestor, ⑬ *Pongo* ancestor, ⑭ Homininae ancestor, ⑮ Hominini ancestor, ⑯ *Pan* ancestor, and ⑰ *Homo sapiens*.

Leveraging a high-resolution primate phylogeny (Fig. 1) and published gene annotations (Shao et al. 2023), we employed a novel computational strategy (TOGA) (Kirilenko et al. 2023) to compile a comprehensive catalog of one-to-one orthologs across 50 primate species and two outgroup species (Malayan flying lemur and Chinese tree shrew) (Fig. 1). From an initial set of 19,270 TOGA-derived orthologous groups, 15,057 were retained for analysis across all 52 species. We excluded 4,213 groups (∼21%) for two primary reasons: missing orthologs in key lineages (*N* = 2,483, suggesting gene loss) or the presence of multi-copy orthologs (*N* = 1,730). The 15,057 protein-coding genes correspond to approximately 78% of the annotated genes in the human reference genome (hg38) and have a combined coding sequence (CDS) length of ∼28.9 Mbp. This method identified 15,057 one-to-one orthologous genes across the 52 species, surpassing the 10,185 genes reported in a previous study (Shao et al. 2023) (Figs. S1 and S2). To compile a comprehensive immune-associated gene catalog for primates, we curated six public datasets (Fig. S3). This process yielded 4,744 one-to-one orthologs across 52 species that were annotated as immune-associated (Fig. S1). Following sequence alignment and trimming, the length distribution of these immune-associated orthologs ranged from 159 to 21,063 bp. This distribution profile remained consistent with the pre-trimming data (Fig. S4), indicating an unbiased data processing pipeline. Our analysis revealed that the average coding sequence length was greater for immune-associated genes relative to other genes (Fig. S5, *P* = 6.7E−08, Wilcoxon rank-sum test).

Studies have identified a correlation between immune response (white blood cell count) and primate mating system (promiscuity), and used primate testis size as an indicator of promiscuity (Nunn et al. 2000; Anderson et al. 2004). Another study shows that mating systems have influenced the evolution of primate immune genes; this pattern is partly due to the increased risk of sexually transmitted infections in more promiscuous species (Wlasiuk and Nachman 2010b). The mating system, social structure, and group size are important components of primate sociality (Swedell 2012), and social condition can alter immune regulation in monkeys (Tung et al. 2012; Snyder-Mackler et al. 2016). In addition, animals can acquire infections—and thus influence immune system evolution—not only through social contacts but also directly from the environment, such as via their diet (Kappeler et al. 2015). In this study, we compiled data on testis weight (indexing mating system), social structure, group size, and diet across 50 primate species. These variables potentially reflect key ecological and social selective pressures relevant to the immune system evolution of primates (Fig. 1 and Table S1). These phenotypes served as key downstream indicators for probing the immune system evolution across primate lineages.

### Evolutionary Modules of Immune-Associated Genes

**Fig. 2.**
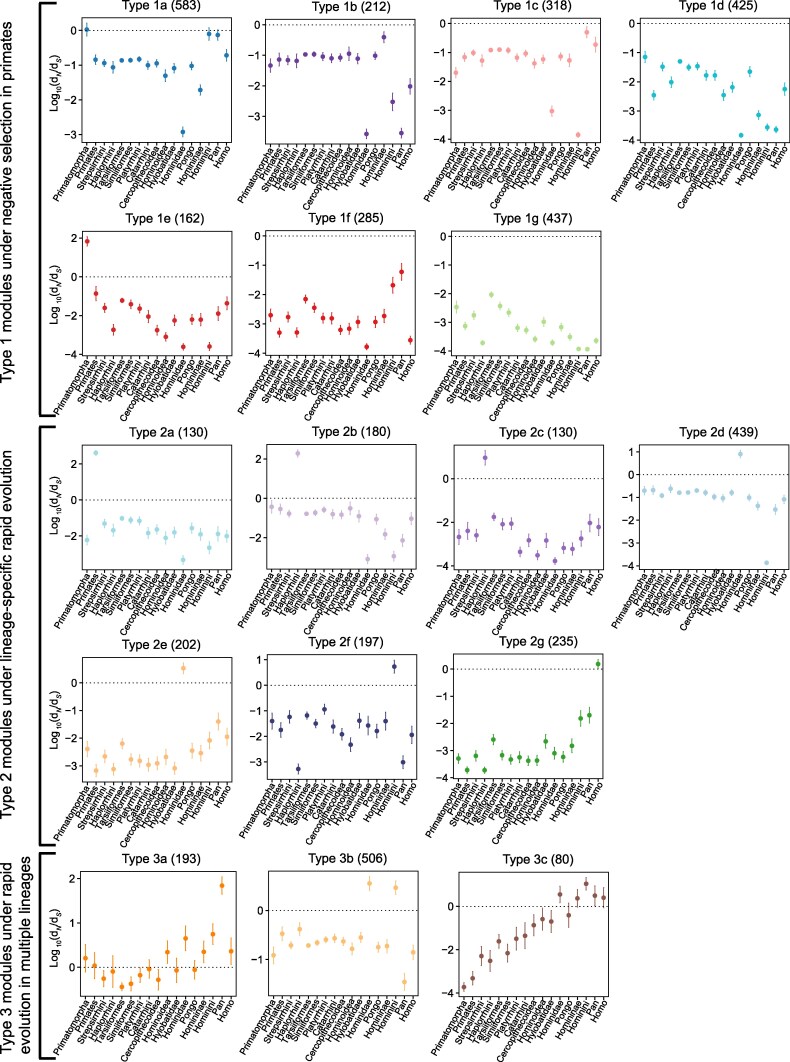
Evolutionary constraint modules of immune-associated genes across 50 primate species. The horizontal axis represents targeted branches. The vertical axis represents Log_10_ (d*_N_*/d*_S_*). The ancestor of Primatomorpha was used as a contrast. The gene number of each module was recorded in parentheses. All modules were inferred by the NbClust (Charrad et al. 2014) and cutree algorithms. The 17 modules were divided into three clusters, including Type 1 modules under negative selection in primates, Type 2 modules under branch-specific rapid evolution in primates, and Type 3 modules under rapid evolution in multiple primate branches.

The ratio of nonsynonymous (d*_N_*) to synonymous (d*_S_*) substitution rates is widely used to quantify the strength and direction of natural selection acting during primate phenotypic evolution. The evolutionary constraint on immune-associated genes is a key factor shaping the immune system diversity across primate lineages. The free-ratio model (PAML) (Yang 2007) allows for the simultaneous estimation of evolutionary pressure on immune-associated genes across every lineage in the primate phylogenetic tree (Fig. 1). This approach enabled us to identify gene modules with similar evolutionary pressure dynamics—a pattern previously unreported. In order to reconstruct the fine-scale evolutionary histories of these immune-associated genes spanning from the common ancestor of all primates to modern humans, we targeted 17 pivotal branches, including Primatomorpha ancestor, Primates ancestor, Strepsirrhini ancestor, Haplorrhini ancestor, Tarsiiformes ancestor, Simiiformes ancestor, Platyrrhini ancestor, Catarrhini ancestor, Cercopithecidae ancestor, Hominoidea ancestor, Hylobatidae ancestor, Hominidae ancestor, *Pongo* ancestor, Homininae ancestor, Hominini ancestor, *Pan* ancestor, and *Homo sapiens*, in this phylogeny (Fig. 1). A matrix of log-transformed d*_N_*/d*_S_* values was created for downstream clustering analysis, comprising 4,744 genes across 17 phylogenetic branches (labeled in Fig. 1). Following screening, 4,714 immune-associated genes with variable evolutionary pressures (d*_N_*/d*_S_* values) across the 17 branches were retained for further analysis (Table S2 and Figs. S6 and S7). Using the NbClust algorithm (Charrad et al. 2014), we identified 17 modules of immune-associated genes (Type 1a–Type 1g, Type 2a–Type 2g, and Type 3a–Type 3c). Despite their distinct identities, all modules showed a similar trajectory of evolutionary pressure across the primate phylogenetic tree (Fig. 2 and Table S3). The modules, which ranged in size from 80 to 583 genes, showed high expression in a tissue- and cell type-specific manner, most notably within the immune system (Fig. S8).

Notably, the log_10_(d*_N_*/d*_S_*) values for seven modules (Type 1a–Type 1g) were consistently negative across all primate lineages, indicating they have been under purifying (negative) selection throughout primate evolution (Fig. 2, Type 1 modules). Functional enrichment analysis revealed that five of these modules (Type 1a–Type 1e) are significantly enriched for genes involved in inflammatory responses (Figs. S9 to S15).

The accelerated evolution that was specific to a particular lineage was identified in seven modules (Type 2a–Type 2g), which were designated as Type 2 modules (Fig. 2). These lineage-specific fast-evolving modules were concentrated on five key evolutionary branches in the primate phylogeny including the common ancestor of all primates, ancestor of Haplorrhini (Old World primates, New World primates, and Tarsiers), ancestor of Hominidae (*Homo*, *Pan*, *Gorilla* and *Pongo*), ancestor of Hominini (*Homo* and *Pan*) and *Homo sapiens* lineage, which have contributed to diverse phenotypic innovations during primate evolution. Two modules within Type 2 (Type 2b and Type 2c) were observed to have undergone Haplorhini-specific rapid evolution. Given their divergent functional enrichment profiles, they may serve distinct immunological roles (Fig. S16). Likewise, two Hominidae-specific fast-evolving modules (Type 2d and Type 2e) exhibited this heterogeneous immunological pattern (Fig. S17). The remaining Type 2 modules (Type 2a, Type 2f, and Type 2g) exhibited divergent immune functions. For example, the primates-specific fast-evolving module (Type 2a) was over-represented in genes involved in GO categories, e.g. chemotaxis (Fig. S18). Hominini-specific fast-evolving module (Type 2f) was enriched in genes involved in GO categories, e.g. T cell proliferation, and *Homo*-specific fast-evolving module (Type 2g) was over-represented in genes involved in GO categories, e.g. T cell receptor signaling pathway and antigen processing and presentation (Figs. S19 and S20).

**Fig. 3.**
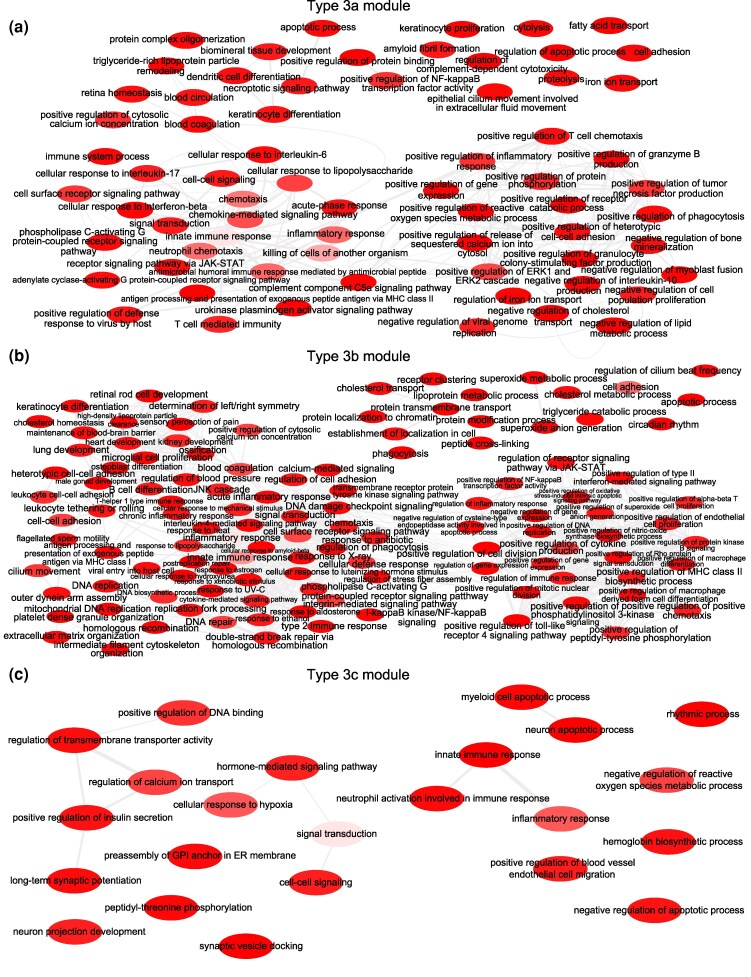
Interactive graph of GO enrichment analysis for type 3 modules. a) Type 3a module. b) Type 3b module. c) Type 3c module. The GO terms with modified *P* value ≤ 0.05 were retrieved from the DAVID (v6.8). The color of the bubble corresponds to the *P* value of the GO term. Lighter colors represent smaller *P*-values. The interactive graph of GO enrichment analysis was retrieved from REVIGO (http://revigo.irb.hr/) and was visualized by Cytoscape (https://cytoscape.org/).

Of particular interest were three modules (Type 3a–Type 3c), which showed accelerated evolution across multiple primate lineages (Fig. 2, Type 3 modules). Across nearly the entire Hominoidea clade, the Type 3a module exhibited a log_10_(d*_N_*/d*_S_*) ratio greater than 0, indicating a history of rapid evolution. Functional enrichment analyses revealed that the Type 3a module was enriched for innate immune response (Fig. 3a), therefore suggesting its important contribution to the evolution of the innate immune system in this clade. Containing up to 506 genes, the Type 3b module exhibited accelerated evolution in the ancestral lineages of both Hominidae and Hominini and showed enrichment in inflammatory response and positive cytokine regulation (Fig. 3b). We also identified a third module (Type 3c) in Type 3 modules, comprising 80 genes. Its evolutionary pressure, as measured by log_10_(d*_N_*/d*_S_*), progressively increased from the common ancestor of all primates to the ancestor of Hominidae, and was further accelerated across the entire Hominidae clade (*Homo*, *Pan*, *Gorilla*, and *Pongo*). Consistently, this module was significantly enriched for genes involved in inflammatory response (Fig. 3c).

As master regulators of gene expression, transcription factors (TFs) control fundamental cellular processes—including differentiation and development—as well as adaptive responses to external perturbations through various signaling pathways (Weidemuller et al. 2021). TF enrichment diverged between modules under purifying selection across all targeted branches (Type 1 modules) and those exhibiting rapid evolution on at least one branch (non-Type 1 modules, encompassing Types 2 and 3). In detail, Type 1 modules showed significantly greater enrichment for TFs relative to non-Type 1 modules (*P* = 6.127e−06, Fisher's Exact Test; Fig. S21 and Table S4). Therefore, we propose that intrinsic immune components like TFs are subject to stronger selective constraints and thus evolve more slowly compared to genes mediating direct host-pathogen interactions.

### Modules Response to Viral Infection

**Fig. 4.**
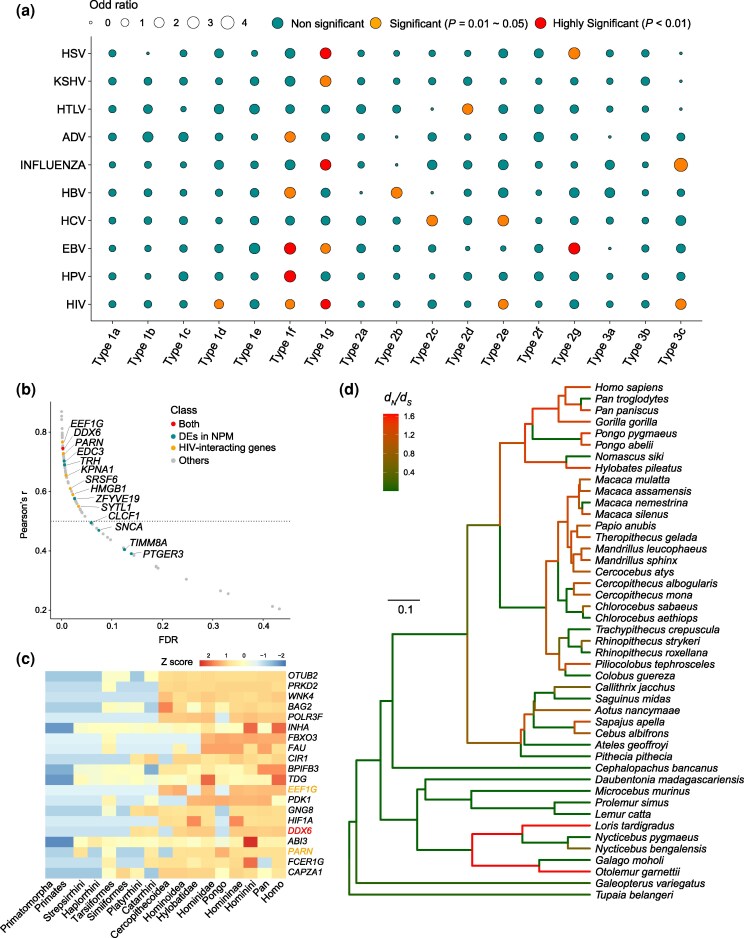
Correlation analyses between modules and virus infection. a) Overlapping significant analysis between infection-related genes to diverse viruses and 17 modules. The *P* values (≤0.05) of overlapping significance were decided by the Fisher Exact Test. HSV: herpes simplex virus. KSHV: Kaposi’s sarcoma herpesvirus. HTLV: human T-lymphotropic virus. ADV: adenovirus. INFLUENZA: influenza virus. HBV: hepatitis B virus. HCV: hepatitis C virus. EBV: Epstein-Barr virus. HPV: human papillomavirus. HIV-1: human immunodeficiency virus type 1. The size of the circle represents the odds ratio estimated in Fisher's Exact Test. b) Member relationship analysis of the Type 3c module. The Pearson r and *FDR* represent the correlations between members and the Type 3c module. The dotted line represents the significant level with *FDR* < 0.05. The yellow dot represents the known HIV-1-interacting genes from a previous study (Enard et al. 2016). The blue dot represents differentially expressed genes in Northern pig-tailed macaque during HIV-1/stHIV-1/SIV infections (Pang et al. 2023). The red dot represents the overlap between HIV-1-interacting genes and differentially expressed genes (Enard et al. 2016; Pang et al. 2023). c) Selective pressure analysis of the top 20 hub genes of the Type 3c module for the targeted branches in the primate phylogeny. The ω values were scaled by the Z-score analysis. d) Visualization of the evolutionary pressure of *DDX6* in the Type 3c module under the primate phylogeny.

While these 17 modules appear central to primate immune system evolution, their roles in the response to pathogenic infection have yet to be determined. Therefore, based on a manually curated database (Enard et al. 2016), we collected 1,256 virus-interacting genes (covering HSV, KSHV, HTLV, ADV, influenza, HBV, HCV, EBV, HPV, and HIV-1) (Fig. S22). We examined the overlap between our modules and these virus-interacting genes. Nine of the 17 modules (Type 1d, Type 1f, Type 1g, Type 2b–Type 2e, Type 2g, and Type 3c) showed significantly high overlap with at least one viral gene set (*P* < 0.05, Fisher's Exact Test; Fig. 4a and Fig. S23), indicating their potential roles in antiviral response.

In detail, the Type 1d, Type 2b, Type 2c and Type 2d was significantly related to HIV-1 infection (*P* = 0.02086, Fisher's Exact Test), HBV infection (*P* = 0.03905, Fisher's Exact Test), HCV infection (*P* = 0.04118, Fisher's Exact Test) and HTLV (*P* = 0.02639, Fisher's Exact Test), respectively (Fig. 4a). Specifically, three modules showed responsiveness to two viral infections: Type 2e to HIV-1 and HCV; Type 2g to EBV and HSV; and Type 3c to HIV-1 and INFLUENZA virus (all *P* < 0.05, Fisher's Exact Test; Fig. 4a). More importantly, two modules (Type 1f and Type 1g), which evolved conservatively under purifying (negative) selection, were implicated in interactions with five distinct viruses, pointing to their core role in multi-pathogen response (Fig. 4a).

Intriguingly, HIV-1 infection was associated with a broader spectrum of modules than the other nine viral infections examined (Fig. 4a). HIV-1 has a recent origin from spills from chimpanzee to human (Gao et al. 1999; Korber et al. 2000; Ghafari et al. 2023) and evolutionary constraints of these modules related to HIV-1 infection were involved in multiple ancestral nodes of primates. Consequently, we speculate that these modules helped mediate the immune response to ancestral lentivirus infections. The Type 3c module, in particular, exhibited progressively increasing evolutionary pressure from the common ancestor of all primates to the human lineage (Fig. 2), implying a long-term coevolutionary arms race between primates and lentiviruses. Further, we analysed the relevance between individual genes and this module by Pearson product-moment correlation analysis and found that 58 genes were significantly correlated to the Type 3c module (Fig. 4b, Cor > 0.5, *FDR* < 0.05). Importantly, seven genes that overlap with known HIV-1-related genes (*EEF1G*, *DDX6*, *PARN*, *KPNA1*, *SRSF6*, *HMGB1*, and *SYTL1*) were also significantly associated with the Type 3c module (Fig. 4b) (Tomescu et al. 2011; Zhu et al. 2011; Gougeon et al. 2012; Reed et al. 2012; Warren et al. 2012; Schaller et al. 2014; Erkelenz et al. 2015; Enard et al. 2016). This convergence strongly suggests the module's functional roles in the lentiviral response. We next examined the overlap between Type 3c module genes and differentially expressed genes (DEGs) from SIV/HIV-1-infected northern pig-tailed macaques (NPMs) (Pang et al. 2023). Among the eight overlapping genes identified, four (50%) were also part of the set significantly correlated with the Type 3c module itself (Fig. 4b). Therefore, our study documented a novel module (Type 3c) that traced the evolutionary arms race between primates and lentiviruses. Significantly correlated members of the Type 3c module (e.g. *DDX6*; Pearson correlation, *FDR* < 0.05) offer key insights into the evolution of primate antiviral immunity, especially against lentiviruses (Fig. 4c and d, and Figs. S24 and S25). Previous studies indicate HIV-1 can use the RNA helicase DDX6 to catalyse capsid assembly (Reed et al. 2012). Thus, our analyses identified molecular determinants for studying coevolutionary interactions between primates and lentiviruses.

### Correlation Between the Evolution of Immune-Associated Genes and Socio-Ecological Phenotypes

Comparative studies indicate that group-living species tend to experience higher pathogen prevalence than solitary species (Tella 2002; Ezenwa 2004). The increased risk of infectious disease transmission is an inherent cost of sociality (Freeland 1976; Kappeler et al. 2015), posing a selective pressure that can drive the evolution of host immune systems. In addition, the relative testis size as an important independent indicator of mating system has been linked to infer molecular immunological variability in primates (Anderson et al. 2004). The intraspecific variation in group size is also correlated to the risk of infection in primates (Nunn and Heymann 2005). Additionally, animals can acquire infections not only through intraspecific traits (e.g. sociality, mating system, group size), but also from the environment (e.g. diet) (Kappeler et al. 2015). Taken together, these variables (Fig. 1a) were considered major indices for exploring the evolutionary drivers of the primate immune system in this study.

**Fig. 5.**
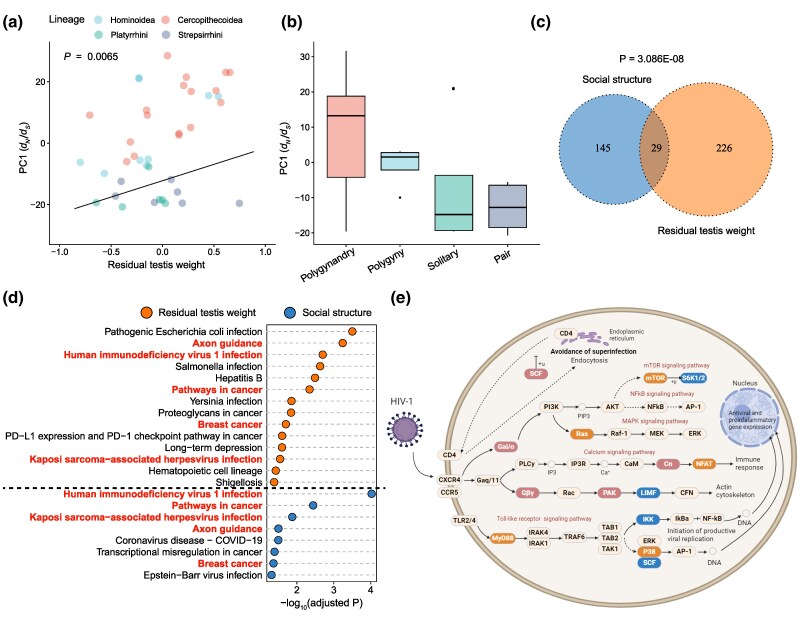
Correlation analyses between d*_N_*/d*_S_* of immune-associated genes and social structure/mating system in primates. a) Correlation analysis between PC1 of d*_N_*/d*_S_* values of immune-associated genes and mating system in primates. b) The PC1 distribution of immune-associated genes for different social structures in primates. c) Venn diagram between immune-associated genes associated with social structure and immune-associated genes associated with the mating system in primates. d) Enrichment analyses of KEGG pathways for immune-associated genes were respectively screened by correlation analyses between d*_N_*/d*_S_* values and mating system/social structure. The highlighted terms in red represent the overlap enriched KEGG pathways associated with the social structure and mating system in primates. e) A significantly enriched signaling pathway—Human immunodeficiency virus 1 infection. The overlapping proteins, respectively identified by social structure and mating system, were highlighted in pink. The unique proteins identified by social structure and mating system were highlighted in blue and orange, respectively.

Here, we collected these traits potentially linked to disease risk in primates, including residual testis size, group size, diet, and social structure, from previously published studies (Table S1). To gain insights into primate immune system evolution, we applied phylogenetic generalized least squares (PGLS) regression (Martins and Hansen 1997). This analysis evaluated the relationships between the selective constraints on immune-associated genes at terminal branches of the primate phylogeny (Fig. 1) and key ecological/life-history variables, while accounting for phylogenetic non-independence. We constructed a matrix of log-transformed d*_N_*/d*_S_* values for all 4,744 immune-associated genes across 50 primate species. After filtering four immune-associated genes harboring the invariable d*_N_*/d*_S_* values across all of the terminal branches (Table S5), the improved matrix of log-transformed d*_N_*/d*_S_* values from 4,740 immune-associated genes across the 50 primate species was used for our downstream analysis. Principal component analysis (PCA) was performed on this improved matrix to capture the variance in d*_N_*/d*_S_* values across one-to-one orthologous immune-associated genes among the terminal species. In the cross-species d*_N_*/d*_S_* analysis, PC1 explained 8.6% of the variance, while other PCs each accounted for less than 3.5% (Figs. S26 and S27). Notably, under the free-ratio model, species from diverse lineages clustered together on PC1, sharing similar scores for selective constraints. This pattern suggests that these immune-associated genes have rather complex evolutionary histories across species. Given that PC1 captured the most variance, we used its scores to examine correlations with several ecological and life-history traits (residual testis mass, social structure, group size, and diet). The maximum-likelihood estimates of Kappa were employed for branch length transformations in this study, with the lowest difference in Bayesian information criterion (dBIC) (Raftery 1995) because other models, posited by larger dBIC values, were worse fitting. In the full model (including social structure, residual testis mass, group size, and diet), Type I ANOVA revealed that social structure and residual testis mass each independently explained a significant portion of the variance in PC1 for evolutionary constraints of immune-associated genes (social structure: sequential sum of squares ANOVA, *P* = 0.019; residual testis mass: sequential sum of squares ANOVA, *P* = 0.0065). In contrast, after phylogenetic correction, neither group size nor diet explained additional significant variation (group size: *P* = 0.78; diet: *P* = 0.73) (Fig. 5a and b, and Table 1 and Table S6). To validate the robustness of our results, we repeated the PGLS analyses after excluding all species with imputed trait values (see Materials and Methods; Tables S6 and S7). The results remained robust and unaffected by data imputation, consistent with our original findings. We further observed that PC2 and PC3 each showed a significant correlation with only one of the two traits—either residual testis mass or social structure (*P* = 0.03469 for PC2 and *P* = 0.03221 for PC3; Tables S8 to S10). In contrast, PC1 exhibited highly significant correlations with both traits (with extremely low *P* values) (Table 1). This supported the conclusion that PC1 captured the largest proportion of variation in evolutionary pressure changes across all 4,740 immune-associated genes, thereby demonstrating the strongest correlation with these immune system phenotypes (residual testis mass and social structure). To identify genes correlated with ecological variables, we employed the same PGLS algorithm and parameters to assess the correlation between the d*_N_*/d*_S_* values (across all terminal branch species) of each of the 4,740 immune-associated genes and these ecological variables. Thus, we identified 174 immune-associated genes with evolutionary correlations to social structure and 255 linked to the o mating system (sequential sum of squares ANOVA, *P* < 0.05). The intersection between immune-associated genes correlated with social structure and those correlated with mating system comprised 29 genes (a statistically significant overlap (Fig. 5c), *P* = 3.086e−08, Fisher's Exact Test), suggesting that mating system and social structure have convergent effects on the evolution of the primate immune system.

**Table 1:** Results for residual testis mass, social structure, group size, and diet models: PC1 ∼ residual testis mass + social structure + group size (log) + diet

Branch length comparisons	dBIC	Weight
**Kappa**	**0**.**0**	**0**.**87**
Delta	5.06	0.069
Lambda	5.13	0.066

Sequential SS ANOVA	Mean sq	*P* value
**Residual testis mass**	**534**.**94**	**0**.**0065**
**Social structure**	**243**.**03**	**0**.**019**
Group size (log)	4.78	0.78
Diet	27.56	0.73
Residuals	62.55	

Model comparisons	dBIC	Weight
Social structure	0.0	0.51
Social structure + Residual testis mass	1.78	0.21
Social structure + Group size (log)	2.95	0.12
Residual testis mass	3.11	0.11
Residual testis mass + Social structure + Group size (log)	5.34	0.035
Residual testis mass + Group size (log)	6.73	0.017
Social structure + Diet	8.93	0.006
Residual testis mass + Diet	10.64	0.002
Residual testis mass + Social structure + Diet	11.02	0.002
Social structure + Group size (log) + Diet	12.48	0.001
Residual testis mass + Group size (log) + Diet	14.29	<0.001
Residual testis mass + Social structure + Group size (log) + Diet	14.67	<0.001

PC1 represents the first principal component of selective pressures of all immune-associated genes across terminal branches in the primate phylogeny (Fig. 1). The Kappa model provides relatively better model fitting than models with other branch length transformations, which is highlighted in bold. Non-Kappa models harbor higher dBIC values and smaller Weight values, indicating that they are not optimal models. Sequential Sum of Squares (SS) ANOVA represents Type I ANOVA of the best model, including all variables. Mean sq: mean squares. The social structure and residual testis mass explain significant amounts of evolutionary constraints of immune-associated genes in primates when excluding effects of phylogenetic signaling, which are also highlighted in bold.

We found that genes associated with social structure and mating system were both enriched in five KEGG pathways including Human immunodeficiency virus 1 infection (hsa05170), Kaposi sarcoma-associated herpesvirus infection (hsa05167), Axon guidance (hsa04360), Pathways in cancer (hsa05200) and Breast cancer (hsa05224) (Fig. 5d). This analysis suggests that the social complexity may shape the primate immune evolution against lentiviruses (e.g. HIV-1/SIV) by elevating the opportunity for transmission in groups. Correlation analyses between immune gene evolution and social/mating traits converged on five proteins in the “Human immunodeficiency virus 1 infection” pathway: SCF, Gαi/o, Gβγ, PAK, and Cn (Fig. 5e). Additionally, we also noted divergent clustering profiles between immune-associated genes related to mating systems and those related to social structure. The immune-associated genes associated with mating system were mainly enriched in pathways related to bacterial infections, e.g. Pathogenic Escherichia coli infection (*P* = 0.0003, modified Fisher's Exact Test), Salmonella infection (*P* = 0.002, modified Fisher's Exact Test), Yersinia infection (*P* = 0.014, modified Fisher's Exact Test) (Fig. 5d). But the immune-associated genes associated with social structure were mainly enriched in pathways related to viral infections (Fig. 5d). For example, eight genes (*RPL13A*, *MAPK8*, *SYK*, *IFNAR2*, *C5AR1*, *RPL35*, *MASP2* and *IKBKG*) were significantly clustered into the Coronavirus disease-COVID-19 pathway (*P* = 0.03, modified Fisher's Exact Test, Fig. S28).

### Evolution of Immune-Associated Genes in NHPs as Disease Models

**Fig. 6.**
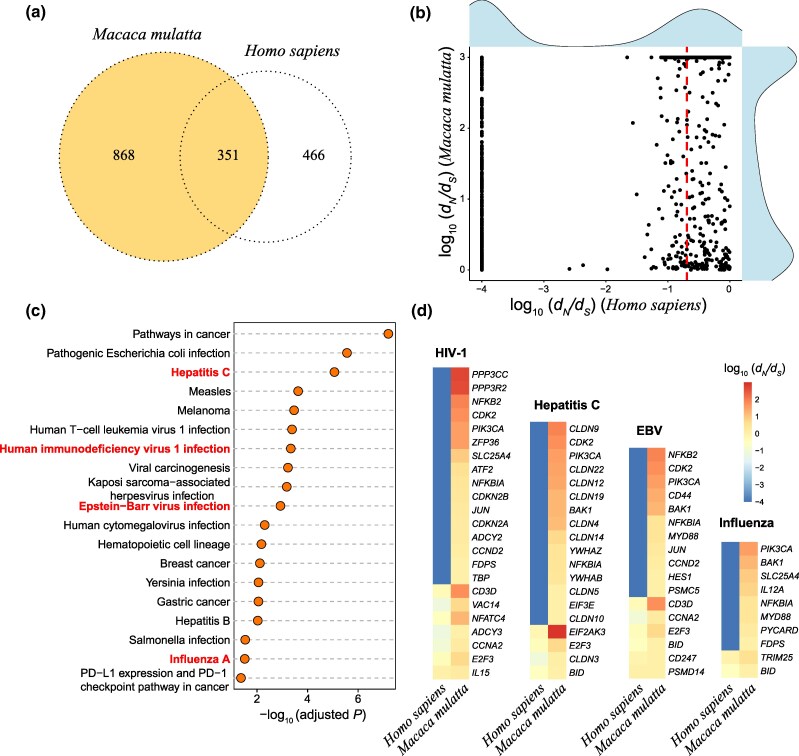
Evolutionary analysis of immune-associated genes in *Homo sapiens* and *Macaca mulatta* lineages. a) Venn diagram of rapidly evolved genes in the *Homo sapiens* lineage and rapidly evolved genes in the *Macaca mulatta* lineage. b) The d*_N_*/d*_S_* values of 868 rapidly evolved genes in *Macaca mulatta* compared with those of the *Homo sapiens* lineage. The red dotted line represents the d*_N_*/d*_S_* value of 0.5 in the *Homo sapiens* lineage. c) KEGG enrichment analyses of the 868 immune-associated genes in the *Macaca mulatta* lineage. The highlighted terms in red indicate the representative disease-related pathways. d) The d*_N_*/d*_S_* values of some rapidly evolved genes in *Macaca mulatta* compared with *Homo sapiens* in the represented disease pathways.

The incidence and severity of diseases are fundamentally shaped by the dynamic interplay between pathogens and the host immune system (Walter et al. 2022; Akbarian et al. 2023). The evolution of the immune system is therefore critical to elucidating disease progression. We hypothesized that lineage-specific rapid evolution on immune-associated genes in nonhuman primates (NHPs) could limit their utility as models for human disease. Compared with the *Homo sapiens* lineage, in the *Macaca mulatta* lineage, 868 immune-associated genes showed unique signatures of rapid evolution (d*_N_*/d*_S_* > 1), as identified under the free-ratio model using the codeml algorithm in PAML4 (Yang 2007) (Fig. 6a and Tables S11 to S13). Among the 868 immune-associated genes under rapid evolution (d*_N_*/d*_S_* > 1) in the *Macaca mulatta* lineage, 85% (739 genes) potentially showed strong purifying selection (d*_N_*/d*_S_* < 0.5) in the *Homo sapiens* lineage (Fig. 6b), suggesting lineage-specific adaptive evolution in macaques.

The functional implications of these 868 genes were subsequently explored using signal pathway enrichment analysis. Surprisingly, these genes were significantly over-represented in pathways involving in diseases, e.g. Hepatitis C (*P* = 8.65E−06, modified Fisher's Exact Test), Human immunodeficiency virus 1 infection (*P* = 4.59E−04, modified Fisher's Exact Test), Epstein-Barr virus infection (*P* = 1.18E−03, modified Fisher's Exact Test), Influenza A (*P* = 0.03, modified Fisher's Exact Test) (Fig. 6c). Of direct relevance, experimental primates (e.g. rhesus and pig-tailed macaques) share susceptibility to important human pathogens, including hepatitis C virus, HIV-1, Zika virus, Ebola virus, and INFLUENZA virus (Agy et al. 1992; Katze et al. 1993; Pang et al. 2017; Estes et al. 2018). Consequently, these species serve as important nonhuman primate models for studying the pathogenesis of human infections. However, we observed divergent evolutionary constraints on genes that were notably enriched in pathways (e.g. HIV-1 infection, hepatitis C, INFLUENZA A) (Fig. 6d and Table S14). Pronounced heterogeneity in selective pressure between rhesus macaque and human was found for key genes in some viral pathways: HIV-1 infection (e.g. *PPP3CC, PPP3R2*), Hepatitis C (e.g. *CLDN9, PIK3CA*), and INFLUENZA A (e.g. *PIK3CA, BAK1*) (Fig. 6d). For example, *PIK3CA* encodes the p110α catalytic subunit of phosphatidylinositol 3-kinase (PI3K) and mutations in *PIK3CA* are known to be involved in a wide range of human cancers (Lai et al. 2015). In this study, *PIK3CA* showed the high divergence in selective constraint between rhesus macaque and human within pathways (e.g. HIV-1, Hepatitis C, INFLUENZA A) (Fig. 6d). These findings indicate that natural selection has shaped divergent immune responses in these species, compromising their translational relevance and necessitating caution in extrapolating comparative results to human medicine.

## Discussion

Owing to their status as our closest living relatives, non-human primates occupy a pivotal position in both biomedical and evolutionary research (Zhang et al. 2019; Warren et al. 2020; Yang et al. 2021). Immunity is a multifaceted defense system that resists pathogen infection, replication, and dissemination. It serves as the critical interface for host-pathogen interactions (Sironi et al. 2015). Nonhuman primate disease models do not fully recapitulate human disease phenotypes and mechanisms, stemming in part from an incomplete understanding of immunity evolution. Previous studies have characterized the phenotypic and genetic diversity of primate immune systems through studies of host susceptibility and pathogen pathogenicity (Barreiro et al. 2010; Hawash et al. 2021). However, the evolutionary mechanisms shaping immune systems across primates remain poorly understood, as most studies have focused narrowly on a limited set of lineages or immune genes. In this study, based on previously released high-quality genomes (Shao et al. 2023), we provided novel insights into the perspective of genes for the evolution of the primate immune system by integrating a new high-powered algorithm—TOGA for obtaining orthologs (Kirilenko et al. 2023) and correlation analyses with immune-related phenotypes.

Prior studies of the primate immune evolution have been constrained by either limited phylogenetic breadth (Nielsen et al. 2005; Barreiro and Quintana-Murci 2010; Cagan et al. 2016) or a narrow focus on specific gene categories (Nakajima et al. 2008; Judd et al. 2021). To overcome these limitations, our study establishes the most complete orthologous catalog of immune-associated genes based on extensive phylogenomic sampling. We then systematically trace the evolutionary pressures acting on these genes across the entire primate phylogeny, encompassing ancestral and terminal branches. Analyses supported our hypothesis that immune-associated genes evolved in a modular fashion during primate evolution. Meanwhile, our analyses revealed high evolutionary and functional heterogeneity among these modules across the primate phylogeny (Fig. 1). This heterogeneity, evident in their sub-classifications and functional enrichment, underscores the evolutionary complexity of the primate immune systems. Nearly 41.2% (7 modules) of total modules exhibited strong signatures of purifying selection and were functionally associated with inflammatory response. Their significant enrichment for transcription factors suggests that these genes may act as stabilizing elements, maintaining evolutionary robustness in the primate immune system. We further assessed the functional relevance of these evolutionary modules in primate responses to pathogen infection. By analyzing their statistical overlap with known virus-interacting genes (Enard et al. 2016), we found that more than half of the modules were implicated, to varying degrees, in viral infection processes. Interestingly, we found that an immune module (Type 3c) was enriched for genes implicated in the response to HIV-1 and may have co-evolved with primate lentiviruses over long evolutionary timescales (Fig. 2). The selective pressure on this module showed a progressive increase from the common ancestor of all primates to that of Hominidae. This pattern suggests a long-term interaction with lentiviruses, as increased pressure was already present in the ancestors of both haplorrhines and strepsirrhines, which diverged approximately 66 million years ago (Shao et al. 2023), further supporting an ancient origin for this evolutionary arms race. Therefore, we inferred the origin of primate lentiviruses may be earlier than the previously estimated ones, e.g. 21 million years ago (Ghafari et al. 2023). Thus, we report an unprecedented case for driving the evolution of primate immune systems.

Diverse factors shape the evolution of the primate immune system (Roecker et al. 1996; O'Sullivan et al. 2013). Consistent with a previous study (Shultz and Sackton 2019), we also used the first principal component (PC1) of d*_N_*/d*_S_* values across genes in species to correlate with these driving factors, although the complex evolutionary histories of immune-associated genes across our extensive phylogenomic samples of primate species may result in a relatively moderate-level assessment by PC1. Through our analysis, we noted that the PC1 of evolutionary constraint in immune-associated genes of primates did not exhibit a significant correlation with dietary patterns, while controlling for the effects of phylogeny. Our results demonstrate that the complexity of social systems (e.g. social structure and mating system) potentially contributes to the ongoing evolution of the primate immune system. Interestingly, group size—a key component of social systems—did not show a significant influence on the evolution of the primate immune system, despite the fact that species with larger populations face a higher risk of infectious disease transmission (Nunn et al. 2015). The evolutionary pressures of a series of immune-associated genes, especially involved in lentivirus infections (e.g. in the HIV-1 infection pathway), showed significant correlations with social structure complexity. Furthermore, several of these immune-associated genes linked to social structure (e.g. *IFNAR2*, *C5AR1*) showed significant enrichment in the Coronavirus disease (COVID-19) pathway (Carvelli et al. 2020; Yaugel-Novoa et al. 2023), suggesting that the evolution of primate sociability may also be relevant to antiviral responses (e.g. against coronaviruses). Moreover, we found that genes associated with the mating system appeared to be involved in bacterial infection, while genes related to social structure were linked to viral infection. Extensive evidence from animal studies has shown that bacterial infections, including those by *Escherichia coli* and *Salmonella enterica*, can impair sperm motility and fertility (Sun et al. 2013; Muntane et al. 2018), suggesting these pathogens may have played a role in shaping mating system evolution. Consequently, the social structure of populations plays an important role in shaping infectious disease dynamics (Silk and Fefferman 2021). The transmission of respiratory viruses is profoundly influenced by host social structure (Shultz and Sackton 2019). Social behaviors such as grooming and the resulting close-proximity contacts (e.g. via airborne droplets) can significantly promote viral transmission. For instance, SARS-CoV-2 spread effectively via intimate social contact. This link renders its transmission dynamics highly dependent on the host's social structure.

In this study, we also identified the differences in selective constraints on immune-associated genes between humans and experimental primates. The large number of genes with high d*_N_*/d*_S_* values in *Macaca mulatta* may reflect lineage-specific selective pressures on the immune system, possibly driven by distinct ecological or pathogen-related factors. Future functional experiments could assess their biological and translational significance.

### Limitations of the Present Study

In order to balance the most comprehensive one-to-one orthologous immune-associated genes among 50 primate species, in this study, we used a log10-transformed d*_N_*/d*_S_* value to decrease potential variances or biases due to some branches potentially with extremely small d*_S_* values, which may result in high d_N_/d_S_ values. Although this method has been used widely (Muntane et al. 2018; Shultz and Sackton 2019), it may not fully rule out the possibility of this impact. And the variance in d*_N_*/d*_S_* across genes cannot be explained with a high percentage by PC1, further suggesting the evolutionary complexity of immune-associated genes in primates. Future methodological advances may allow for a direct validation of our findings. Furthermore, despite the reasonable assumption that conserved one-to-one orthologs maintain similar functions, our approach cannot definitively rule out that some genes may lack immune-related roles in specific non-human primate lineages. Thus, the human-centric gene annotations may bias our identification in immune-associated genes and may also miss the immune-associated genes in specific non-human primate lineages, which are not annotated as potential immune-associated genes in human lineage. Furthermore, the intensity of selective pressure acting on different primate lineages (e.g. those within the Type 3a module, such as *Pan*) may partly challenge assumptions regarding the evolutionary conservation of these gene functions. While the terminal branches of the primate species tree presented in Fig. 1 were not the primary focus of this study—as our aim was to trace the evolution of immune system complexity from key ancestral nodes in primates to the human lineage—these extant lineages remain important for future investigations aimed at comprehensively understanding primate immunity. This study represents an initial step toward understanding the complex evolutionary histories of immune-associated genes across diverse primate species. We hope that future studies, particularly functional experiments across diverse primate lineages, will address these gaps and overcome the biases of human-centric gene annotations in comparative primate genomics.

## Materials and Methods

### Manual Collection of Immune-Associated Genes

We manually collected a total of 5,635 human protein-coding genes with known immune functions from six public resources: Gene Ontology (GO) (Gene Ontology Consortium et al. 2023), Kyoto Encyclopedia of Genes and Genomes (KEGG) (Kanehisa and Goto 2000), InnateDB (Breuer et al. 2013), ImmPort (Bhattacharya et al. 2018), Human Phenotype Ontology (HPO) (Gargano et al. 2024), and a previously published catalog of virus-interacting proteins (VIPs) (Enard et al. 2016). Specifically, we compiled immune-associated genes from multiple sources: 1,318 genes from InnateDB, 1,355 genes from ImmPort, 2,510 genes from the Gene Ontology (GO) database, 1,189 genes from KEGG, 1,938 genes from HPO, and 1,234 genes from virus-interacting proteins (VIPs). Immune-associated genes from the GO, KEGG, HPO, InnateDB, and ImmPort databases were retrieved and immune-related categories were listed in Tables S15 to S19. The overlap among databases was visualized using the upsetR package (Conway et al. 2017), and all retrieved genes were merged into a non-redundant immune-associated gene catalog for downstream analyses.

### Identification of One-to-One Orthologous Genes in Primates

Whole genome sequences from 52 species (Fig. 1a), comprising 50 primate species and two outgroup species (*Galeopterus variegatus* and *Tupaia belangeri*), were analysed in this study. Orthologous genes in each genome were detected using the TOGA pipeline (Kirilenko et al. 2023), with the human genome (hg38) as the reference. TOGA implemented whole-genome alignments to infer orthologous loci and enhance the annotation of conserved genes and ortholog detection (Kirilenko et al. 2023). Initially, genome alignment chains were created for each species using the human genome (hg38) as a reference through the make_lastz_chains pipeline (https://github.com/hillerlab/make_lastz_chains) (Kirilenko et al. 2023). Subsequently, the TOGA software was employed to infer orthologous genes based on the human GENCODE V38 (Ensembl 104) gene annotation. Homologous groups (HOGs) across 52 species were then constructed based on the inferred orthologous relationships from the TOGA software. Only the longest transcript was considered for each gene locus when multiple splicing variants were present. Entire HOGs were excluded if they contained fewer than two sequences in any main taxonomic group (i.e. Homininae, Hylobatidae, Cercopithecinae, Colobinae, Platyrrhini, Tarsiiformes, and Strepsirrhini) or more than three sequences for any species. The longest sequences were retained for species with multiple homologous sequences. This produced a comprehensive set of HOGs, which we used in the following analyses.

### Alignment and Filtering of Orthologous Genes in Primates

Alignments of these HOGs were generated using an adapted pipeline of the Selectome (v4) (Proux et al. 2009). Initially, the protein alignments for each HOG were created using MAFFT with the “L-INS-I” algorithm (Katoh and Standley 2013). The MaxAlign v1.1 algorithm (Gouveia-Oliveira et al. 2007) was then applied to identify and remove poorly aligned sequences (i.e. gap-rich sequences) from the multiple sequence alignments. Protein sequences, identified by MaxAlign and their deletions, ensuring that at least two sequences remained in the main taxonomic groups (see above), were discarded. The remaining protein sequences of each HOG were realigned using MAFFT with the “L-INS-I” algorithm, and residues with a low transitive consistency score (TCS; lower than 6) in alignments were masked using M-Coffee (Wallace et al. 2006). Finally, these protein alignments were reverse-transformed to nucleotide alignments by applying seq_reformat in the T-Coffee package (Notredame et al. 2000).

### Evolutionary Dynamics of Immune-Associated Genes in Primates

We aimed to characterize the signature of selective pressure of immune-associated genes across the primate genomes. An estimate of the d*_N_*/d*_S_* in each branch of the primate phylogeny was obtained for each gene using the free ratio model from PAML (v4.9) (Yang 2007). In our study, one of our purposes is to estimate the immunity evolution of key evolutionary nodes from the most recent common ancestor of all primates leading to the human lineage. Therefore, we included the 16 internal branches and the human lineage as the representative key evolutionary branches in the whole primate phylogeny (Fig. 1). This approach allowed us to capture the most representative lineage-specific selective dynamics while reducing noise from terminal branches. Subsequently, a matrix of log-transformed d*_N_*/d*_S_* values was created for 17 targeted primate branches (spanning the primate phylogeny; refer to Fig. 1) at immune-associated genes. We used the log-transformed d*_N_*/d*_S_* values to reduce the influence of extreme values. Such log-transformation has been shown to improve robustness and comparability of d*_N_*/d*_S_* values in previous evolutionary studies (Muntane et al. 2018; Shultz and Sackton 2019). Genes exhibiting constant d*_N_*/d*_S_* values across all branches of the primate phylogeny were excluded from the subsequent analysis. A gene was defined as having a constant d*_N_*/d*_S_* if its estimated d*_N_*/d*_S_* values were identical across all targeted branches of the primate phylogeny. We performed the NbClust testing (“NbClust” package in R, index = “all”) to determine the optimal number of clusters (Charrad et al. 2014). Hierarchically clustering analysis was then conducted on the d*_N_*/d*_S_* value matrix, using basal R functions “dist” with the Euclidean distance and “hclust” with the ward.D2 method. Subsequently, gene modules in the d*_N_*/d*_S_* values matrix were identified using the “cutree” function with k = 17. Changes in the d*_N_*/d*_S_* values of gene modules throughout primate evolution were visualized using ggplot2 in R. Functional enrichment analyses (GO, KEGG, and tissue expression) were performed for each gene module separately, using the online DAVID platform (v6.8) (Dennis et al. 2003). Significantly enriched terms were identified using the modified *P*-values of ≤0.05. The interactive graphs and treemaps of GO enrichment for modules were retrieved from REVIGO (http://revigo.irb.hr/) and visualized using Cytoscape v3.10.1 (https://cytoscape.org/). A comprehensive list of human transcription factors was obtained from the SCENIC database (https://raw.githubusercontent.com/aertslab/SCENICprotocol/master/example/allTFs_hg38.txt). The Fisher's Exact Test was used to assess the overlapping significance between each gene module and the set of transcription factors.

### Potential Roles of Immune Modules in Adaptation of Primates Against Viruses

To investigate potential roles of immune modules in adaptations of primates against viruses, we conducted Fisher's Exact Tests to assess the significance of overlap between virus-interacting proteins (Enard et al. 2016) and gene modules identified in our study. Here, we performed one-tailed Fisher's Exact Tests with a specific parameter (alternative = “greater”) in R software for each module to evaluate whether the observed overlap was significantly enriched with a higher proportion relative to a random expectation. Subsequently, we identified the core genes (*FDR* ≤ 0.05) in the Type 3c module, which exhibited a significant overlap with HIV-interacting proteins (Enard et al. 2016). Using the basal R function “cor.test”, we calculated the Pearson Correlation Coefficient to examine the correlations between the evolutionary pressure of each immune gene and the overall evolutionary pressure of the module. We applied the *FDR* procedure (Benjamini and Hochberg 1995) to correct the corresponding *P*-value (in the correlations) for multiple tests using the stats package with the p.adjust function. Genes with *FDR*-corrected *P*-values of ≤ 0.05 were considered as the core genes. The heatmap of d*_N_*/d*_S_* values across the primate phylogeny for the top 20 core genes in the Type 3c module was generated using the R package pheatmap. The differentially expressed genes of Northern pig-tailed macaques in response to HIV-1, SIV, and stHIV infections at both acute and chronic stages (1, 2, 3, 4, 5, 6, 8, 12, 18, and 24 wk after infection) were retrieved from a previous study (Pang et al. 2023). The correlation between Pearson's r and *FDR* of members in the Type 3c module was visualized using ggplot2 with highlighting differentially expressed genes and virus-interacting proteins (Enard et al. 2016). The d*_N_*/d*s* values of the gene *DDX6* in the primate phylogeny were visualized using the ggtree package (Yu et al. 2017) in R.

### Correlation Between Evolutionary Pressure of Immune-Associated Genes and Complex Traits in Primates

Ecological and behavioral traits are only well-characterized for extant species because ancestral phenotypes remain largely unknown. Therefore, we focused the phenotype–genotype association analysis on extant species to ensure biological interpretability and data reliability. To achieve this, we collected the species traits potentially linked to immunity evolution in primates, including residual testis size, group size, diet and social structure, from published references (Table S1). For the information on these traits was not available for some species, we imputed missing values based on the average values from other species in the same genus. Prior to analysis, group size was log-transformed. Residual testis size was calculated as the residuals of the regression of log-transformed testis size versus log-transformed body size from a linear regression model, fitted using the “lm” function in R. Subsequently, we created a matrix of the log-transformed d*_N_*/d*_S_* values at immune-associated genes across species. We filtered out immune-associated genes using a threshold of more than five species with missing values per gene. Missing values in the matrix were imputed using the non-linear iterative partial least squares algorithm in the “nipals” R package (Andrecut 2009). Principal components analysis (PCA) was performed on this matrix using the “prcomp” function in R to summarize the variance of d*_N_*/d*_S_* values across genes and species. We subsequently assessed whether PC1, explaining the variance in d*_N_*/d*s* values across species, correlated with these primate traits (above). Phylogenetic generalized least squares (PGLS) analysis was applied to test the correlations using the “pgls” command from the caper package (Orme et al. 2025), with maximum-likelihood estimations of Lambda, Kappa, and Delta for branch length transformations. Specifically, Lambda (λ) scaled the internal branches of the tree and reflects the strength of phylogenetic signal (λ = 1 indicates strong phylogenetic correlation, whereas λ = 0 implies none). Delta (δ) transformed the sum of the shared branch lengths between species by raising them to the power of δ, capturing shifts in the evolutionary rate over time (values <1 suggest early bursts, and >1 indicate recent acceleration). Kappa (κ) adjusted the scaling of all branch lengths in the phylogeny by raising them to the power of κ. As κ decrease towards zero, all branches will become of equal length, representing an evolutionary model where variation accumulates only when two clades diverge. Model comparisons were conducted using BIC, which is the most parsimonious model and uses a more conservative penalty for additional terms. Sequential analysis of variance (Type I ANOVA) was used to identify variables that explained a significant amount of PC1 variation.

### Identification of Immune-Associated Genes Correlated With Special Traits in Primates

To identify the genes and molecular functions contributing to the correlation between PC1 and residual testis size, as well as social structure (as described in the Results section), we calculated the *P*-value for the associations between log-transformed d*_N_*/d*_S_* values and residual testis size or social structure for each gene individually. PGLS analysis was performed for each gene using the “pgls’ function from the caper package (Orme et al. 2025), with Kappa transformations (best model; see Results). Because the PGLS method has a strong power to calculate the relationship between genes and phenotypes under a phylogeny, we reported unadjusted *P*-values to interpret genes with *P* < 0.05 as candidates for further investigation. Venn diagrams were constructed to depict the number of shared genes showing correlation with residual testis size and social structure. Fisher's Exact Test was applied to determine the significance of overlapping in genes showing correlation with residual testis size and social structure. Additionally, KEGG pathway enrichment analyses were performed for genes correlating with residual testis size or social structure separately, using the online platform DAVID (v6.8) (Dennis et al. 2003). Significantly enriched KEGG pathways were identified with modified *P*-values of ≤ 0.05.

### Rapidly Evolved Genes in *Macaca mulatta* Compared With *Homo sapiens*

To identify immune-associated genes uniquely under rapid evolution in the *Macaca mulatta* lineage compared with the *Homo sapiens* lineage, we calculated the genes with d*_N_*/d*_S_* values exceeding 1 both in *Macaca mulatta and Homo sapiens*. The venn diagram was constructed to depict the number of shared rapidly evolved genes between *Macaca mulatta* and *Homo sapiens*. A scatter plot showing the comparisons of log-transformed d*_N_*/d*_S_* values between *Macaca mulatta* and *Homo sapiens* for the rapidly evolved genes uniquely in *Macaca mulatta* was created using ggplot2 in R. Subsequently, KEGG pathway enrichment analysis was performed via the online DAVID platform (Dennis et al. 2003), with significantly enriched pathways identified using the *FDR*-corrected *P*-values of ≤ 0.05. The *FDR* in DAVID requests adaptive linear step-up adjusted *P*-values for approximate control of the false discovery rate, as discussed in a previous study (Benjamini and Hochberg 2000). Additionally, a heatmap presenting the log-transformed d*_N_*/d*_S_* values in *Macaca mulatta* and *Homo sapiens* for rapidly evolved genes uniquely in *Macaca mulatta* was generated using the R package pheatmap.

## Supplementary Material

evag087_Supplementary_Data

## Data Availability

The Catalog of novel homologous genes, including 1,5057 items among 52 species, the newly identified immune-associated set, and sequences of all orthologous genes in this study were deposited at the public Dryad dataset (https://doi.org/10.5061/dryad.6q573n65z). We uploaded scripts for identifying orthologous genes, calculating d*_N_*/d*_S_* values, and performing the downstream statistical analyses to a public GitHub repository with the configuration files, example input data, and user-specified arguments (https://github.com/zhangxp1993/Immune-associated-orthologous-genes-across-primates). The primate species phylogeny in Newick format was submitted to the Dryad database (https://doi.org/10.5061/dryad.6q573n65z). Immune annotation categories involved in GO, KEGG, HPO, InnateDB, and Immport were described in Tables S15 to S19.
