# Supplementary material for: Evolutionary Complexity of Primate Immune System Uncovered by the Extensive Phylogenomic Sampling
Source: Genome Biol Evol. 2026 Apr 1;18(4):evag087. doi: 10.1093/gbe/evag087 (PMC13089530; doi:10.1093/gbe/evag087)
Supplement: evag087_Supplementary_Data [file evag087_supplementary_data.zip › Supplementary Materials (20260327).docx]

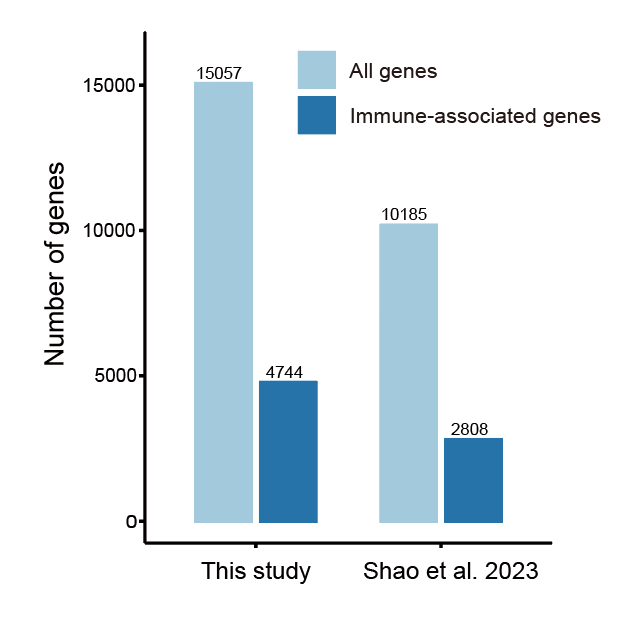


**Supplementary fig 1. Comparison of identified one-to-one orthologous genes and immune-associated genes between this study and a previous study across 50 primate species.** This study identified more one-to-one orthologous immune-associated genes than that of a previous study (Shao et al. 2023)


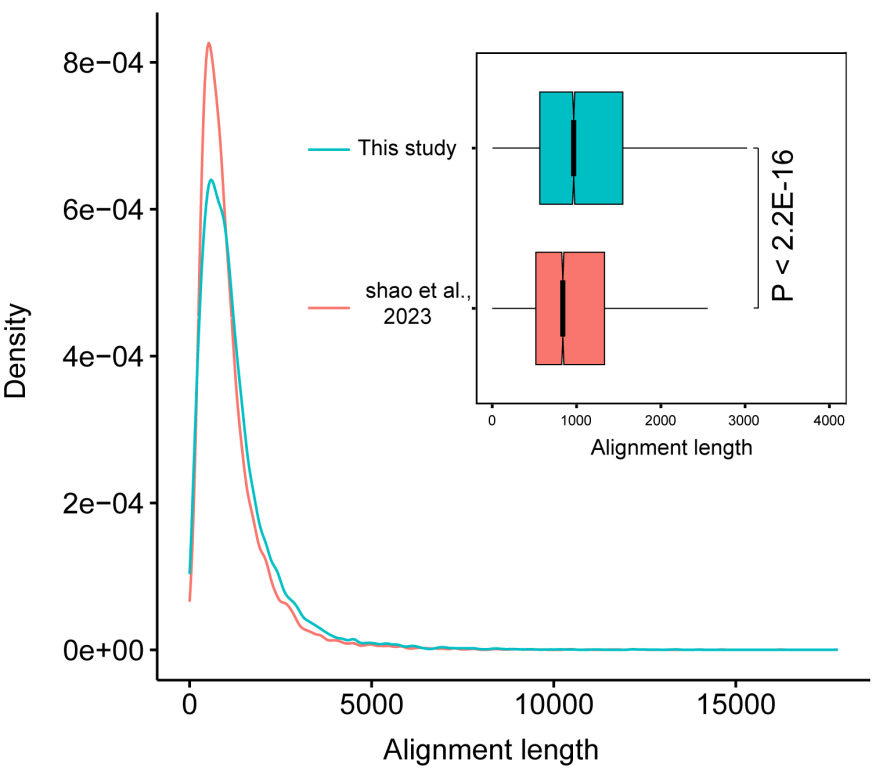


**Supplementary fig 2. Comparison of alignment length of one-to-one orthologous coding sequences after trimming between this study and a previous study.**

**
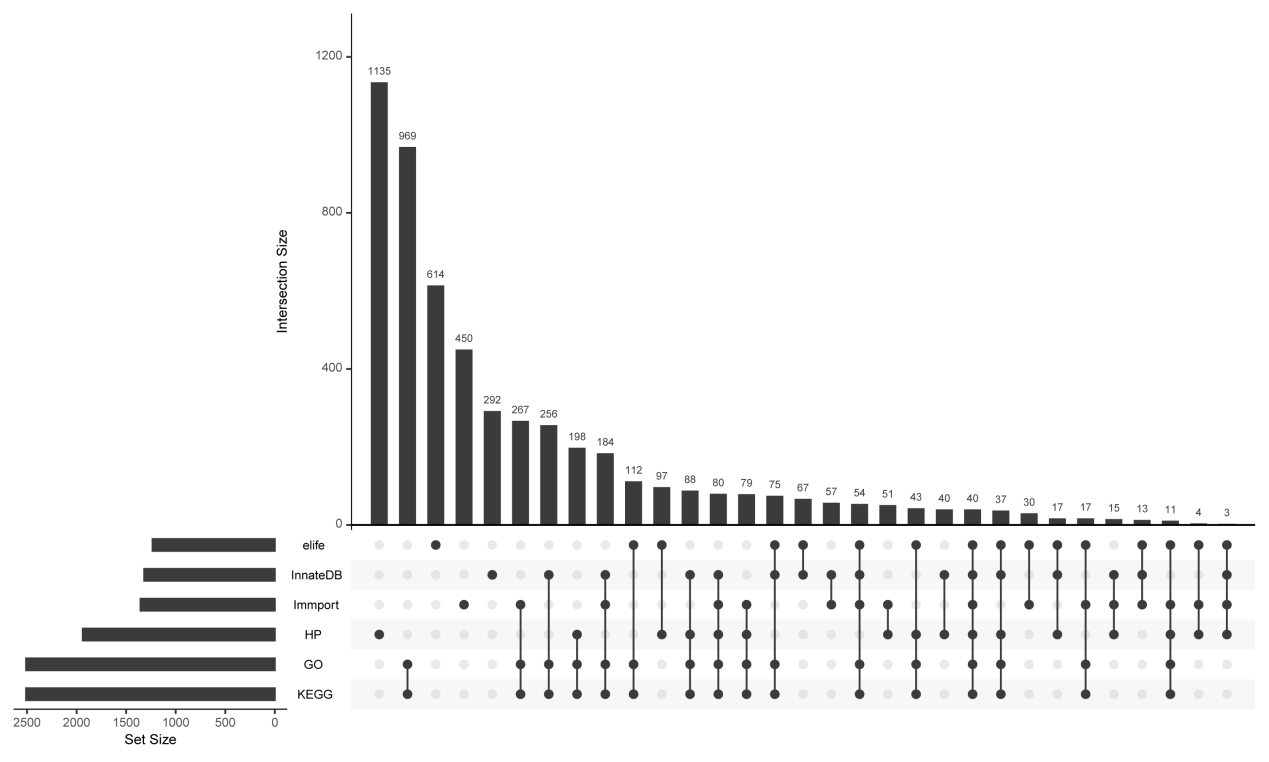
**

**Supplementary fig 3. Identification of immune-associated genes from six public databases.** The intersection size of immune-associated genes between different databases were shown in this figure.


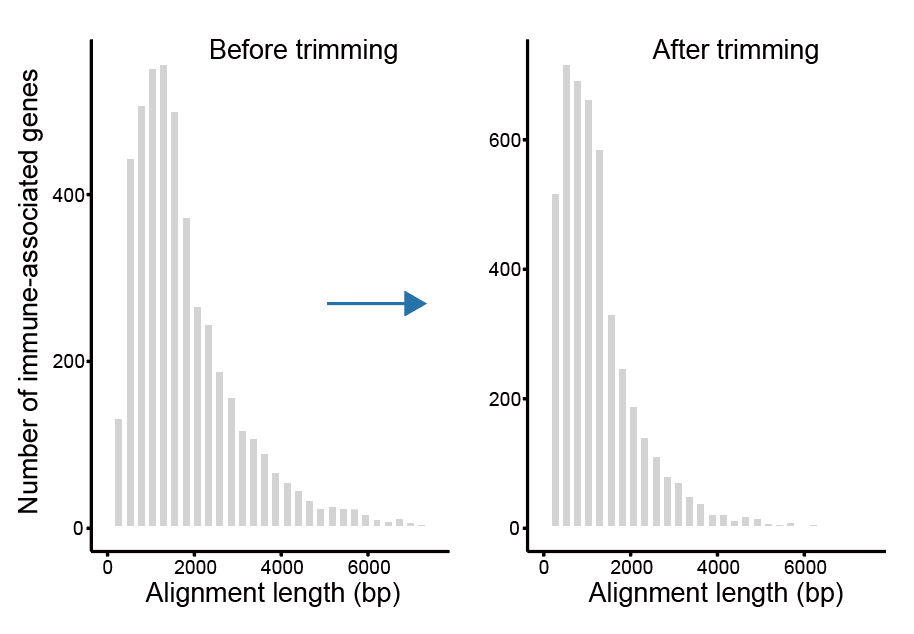


**Supplementary fig 4. Sequence length distribution of one-to-one orthologous immune-associated genes in primates before and after sequence trimming.**

**
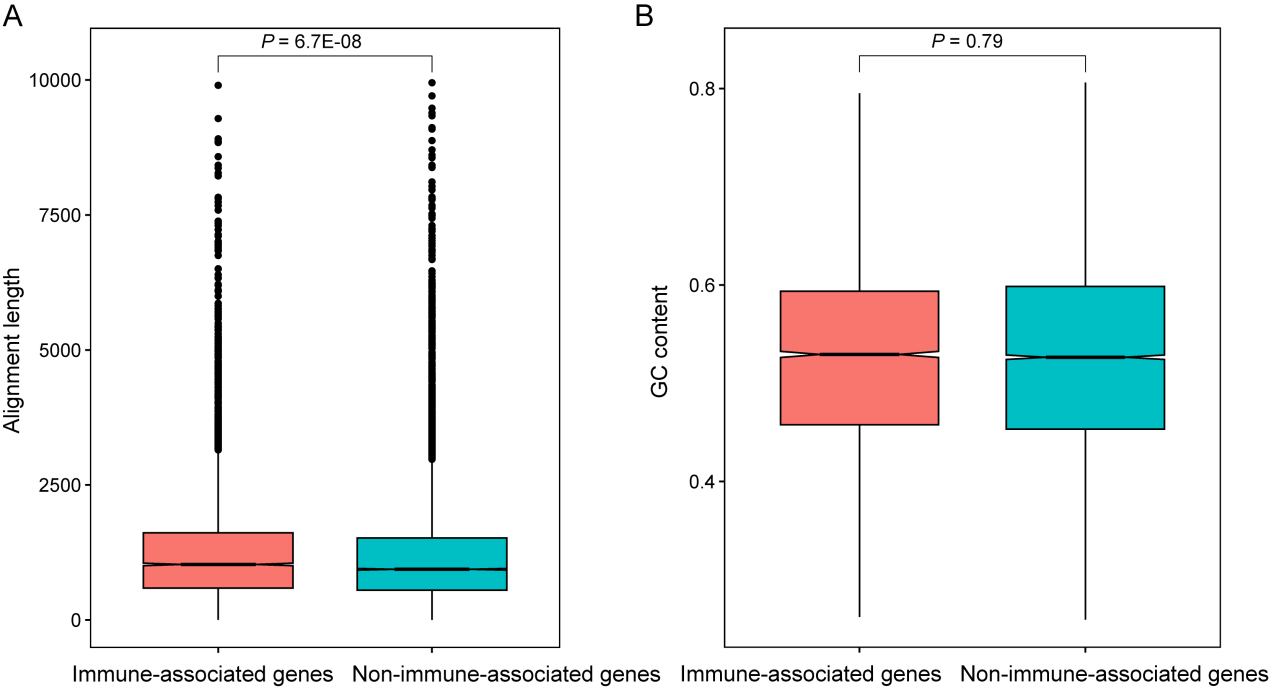
**

**Supplementary fig 5. Comparative analyses of evolutionary features between immune-associated genes and non-immune-associated genes in primates.** The statistics significance was calculated by Wilcoxon Rank Test.


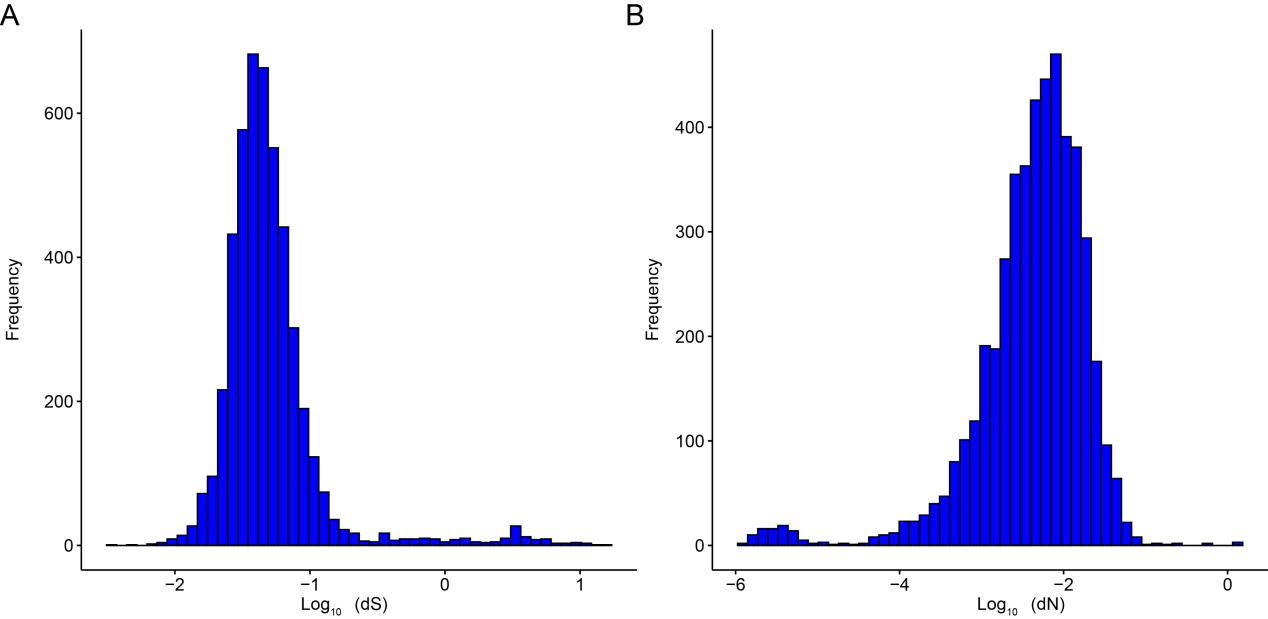


**Supplementary fig 6. The distributions of d*_S_* and d*_N_* values of all one-to-one orthologous immune-associated genes across 17 branches in a primate phylogeny.** (A) The distribution of d*_S_* values of all one-to-one orthologous immune-associated genes across 17 branches in a primate phylogeny. (B) The distribution of d*_N_* values of all one-to-one orthologous immune-associated genes across 17 branches in a primate phylogeny. The x-axis represents log_10_ (mean d*_N_* or mean d*_S_*) for each of all one-to-one orthologous immune-associated genes across 17 branches in the primate phylogeny. The y-axis represents the frequencies of these genes.


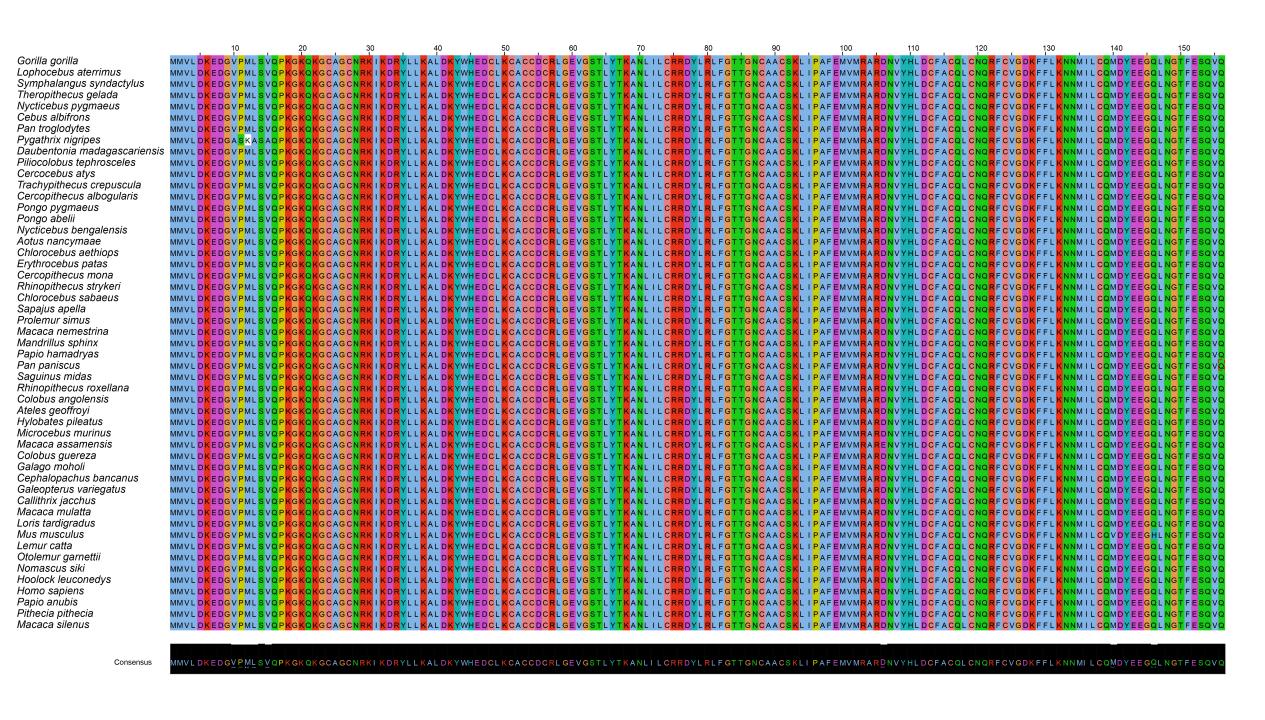


**Supplementary fig 7. An example of protein sequence alignments for *LMO1* without variation of d*_N_*/d*_S_* values across 17 targeted branches in the primate phylogeny.**


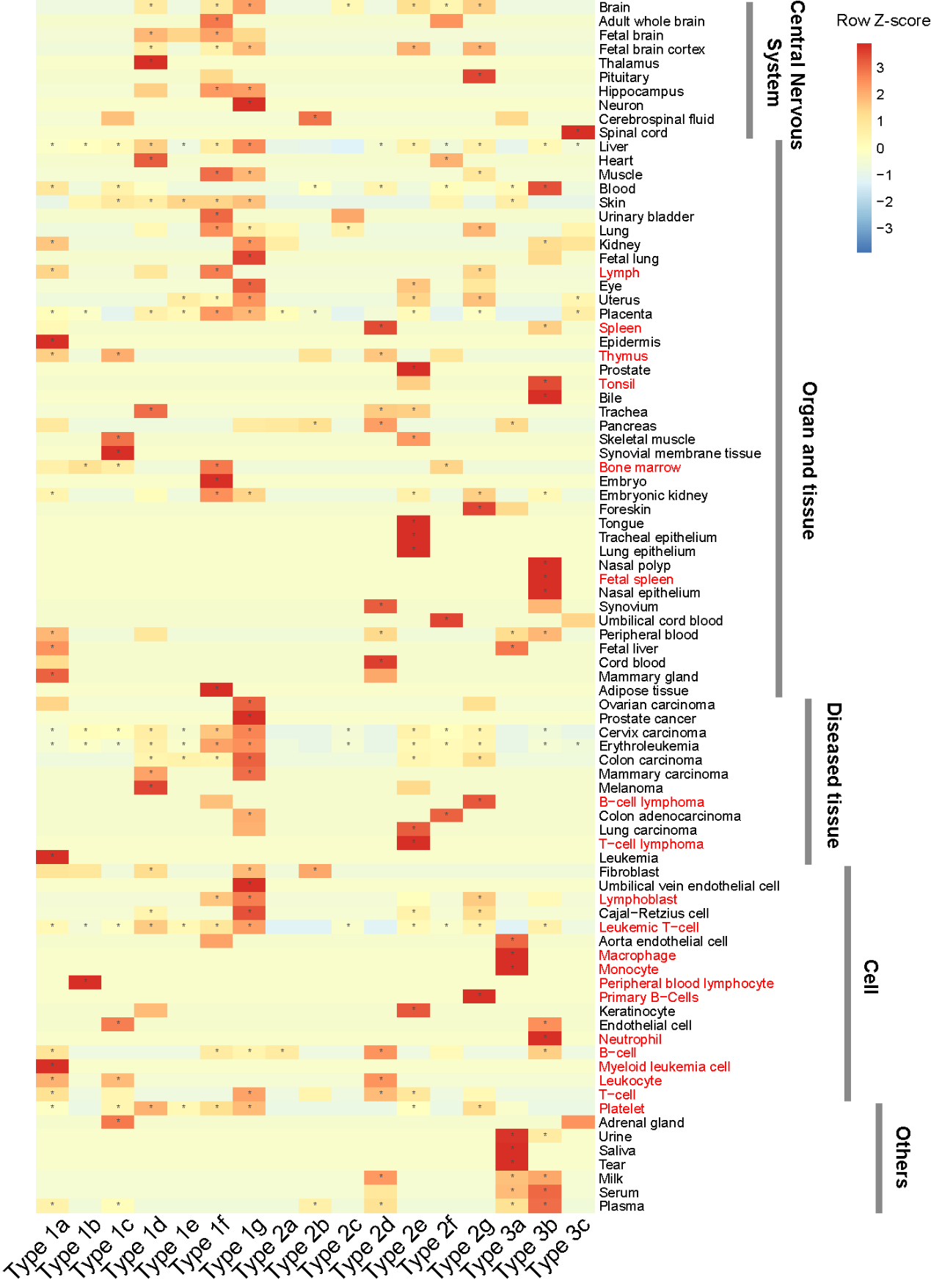


**Supplementary fig 8. Enrichment analyses of tissue-specific high expression for 17 modules in primates.** Significance was obtained from the David v6.8 functional annotation pipeline.


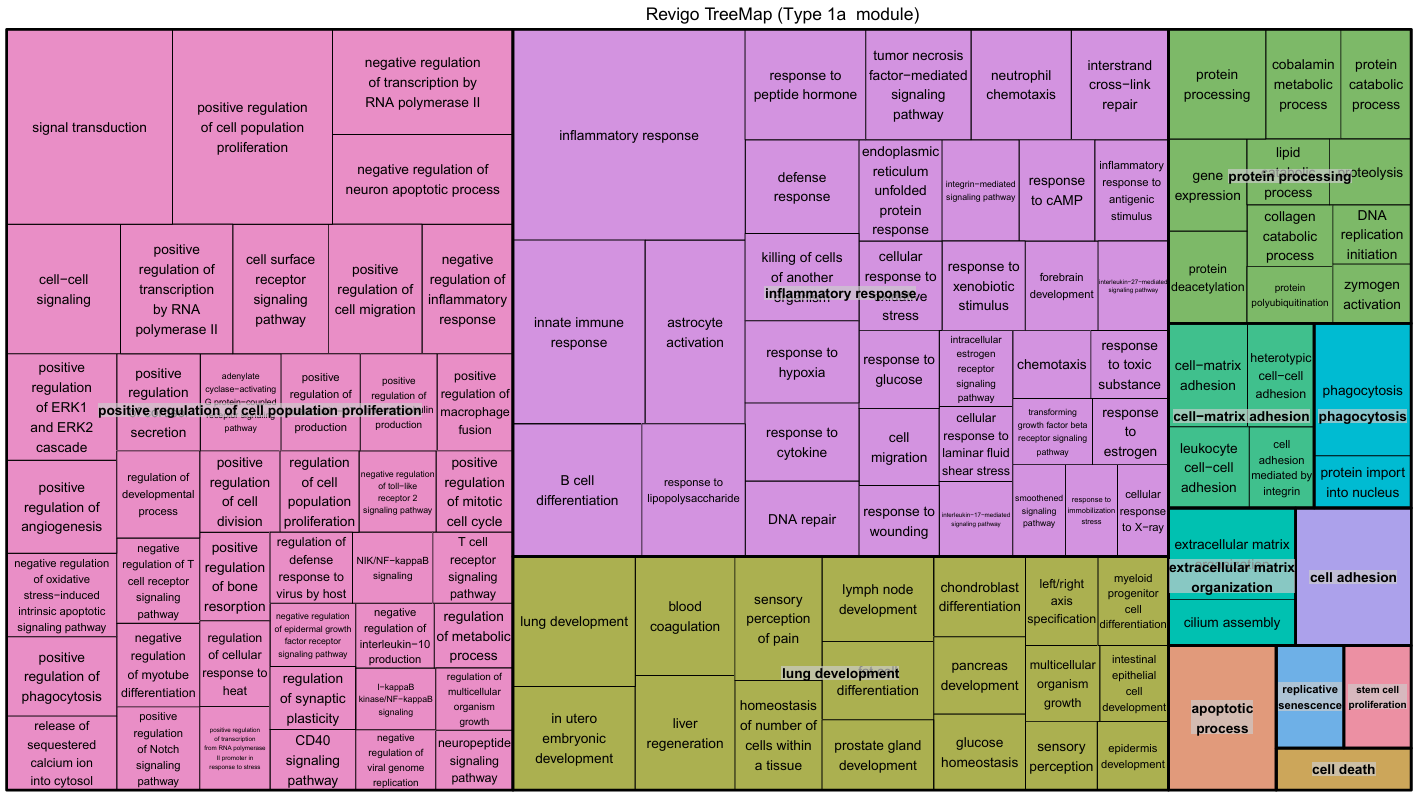


**Supplementary fig 9. Revigo treemap of significant GO enrichment terms for the Type 1a module.** The significant GO enrichment terms with *P* value ≤ 0.05 were identified by DAVID v6.8 pipeline.


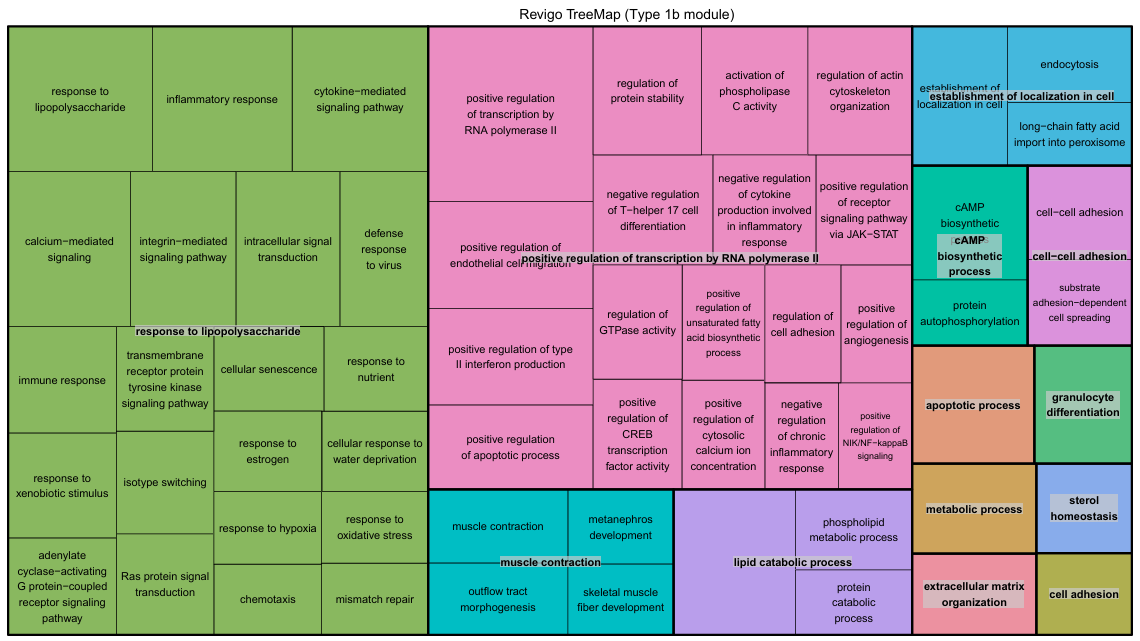


**Supplementary fig 10. Revigo treemap of significant GO enrichment terms for the Type 1b module.** The significant GO enrichment terms with *P* value ≤ 0.05 were identified by DAVID v6.8 pipeline.


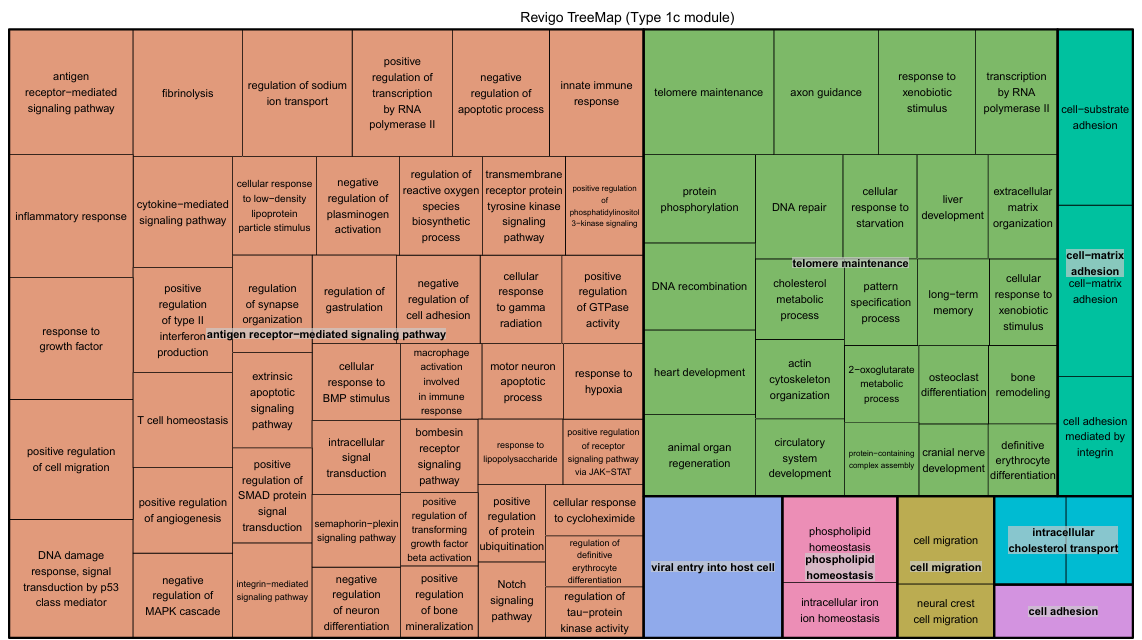


**Supplementary fig 11. Revigo treemap of significant GO enrichment terms for the Type 1c module.** The significant GO enrichment terms with *P* value ≤ 0.05 were identified by DAVID v6.8 pipeline.


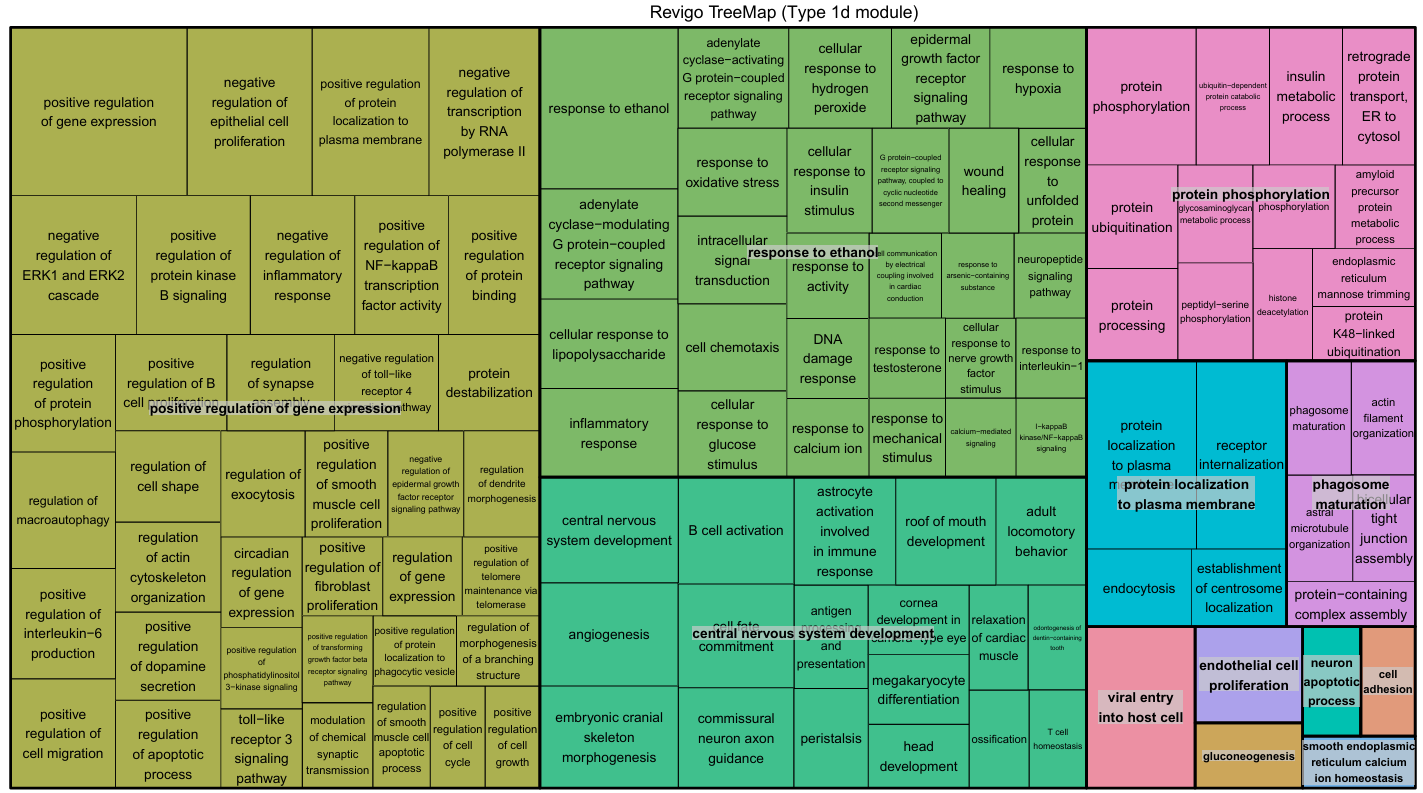


**Supplementary fig 12. Revigo treemap of significant GO enrichment terms for the Type 1d module.** The significant GO enrichment terms with *P* value ≤ 0.05 were identified by DAVID v6.8 pipeline.


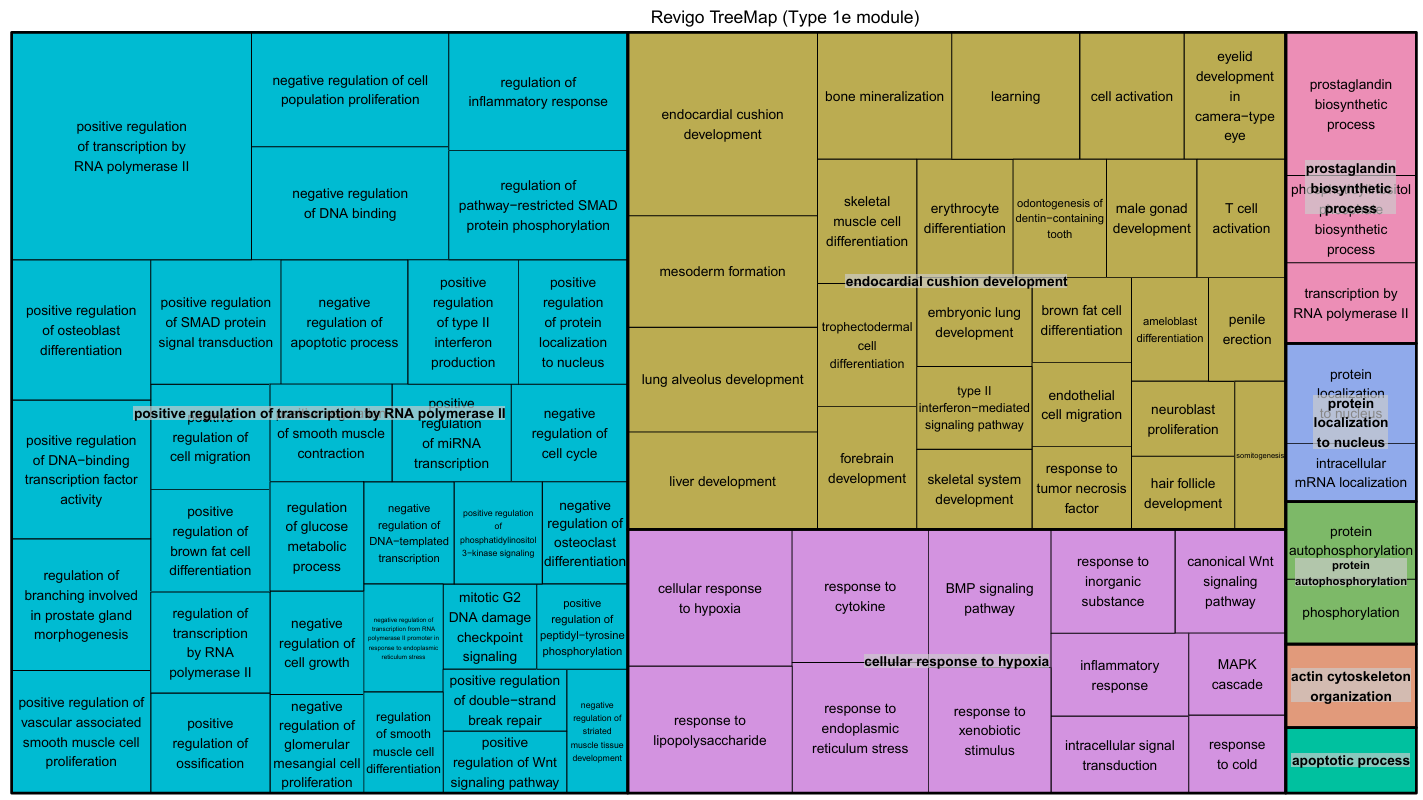


**Supplementary fig 13. Revigo treemap of significant GO enrichment terms for the Type 1e module.** The significant GO enrichment terms with *P* value ≤ 0.05 were identified by DAVID v6.8 pipeline.


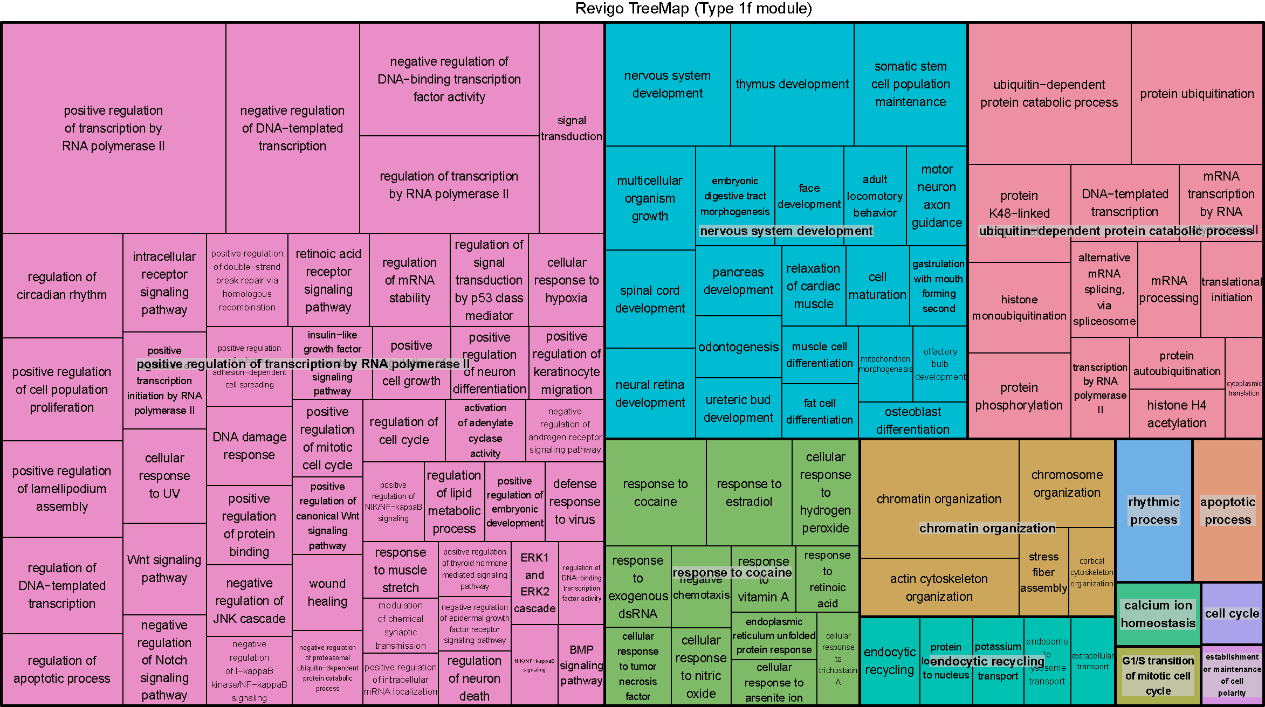


**Supplementary fig 14. Revigo treemap of significant GO enrichment terms for the Type 1f module.** The significant GO enrichment terms with *P* value ≤ 0.05 were identified by DAVID v6.8 pipeline.


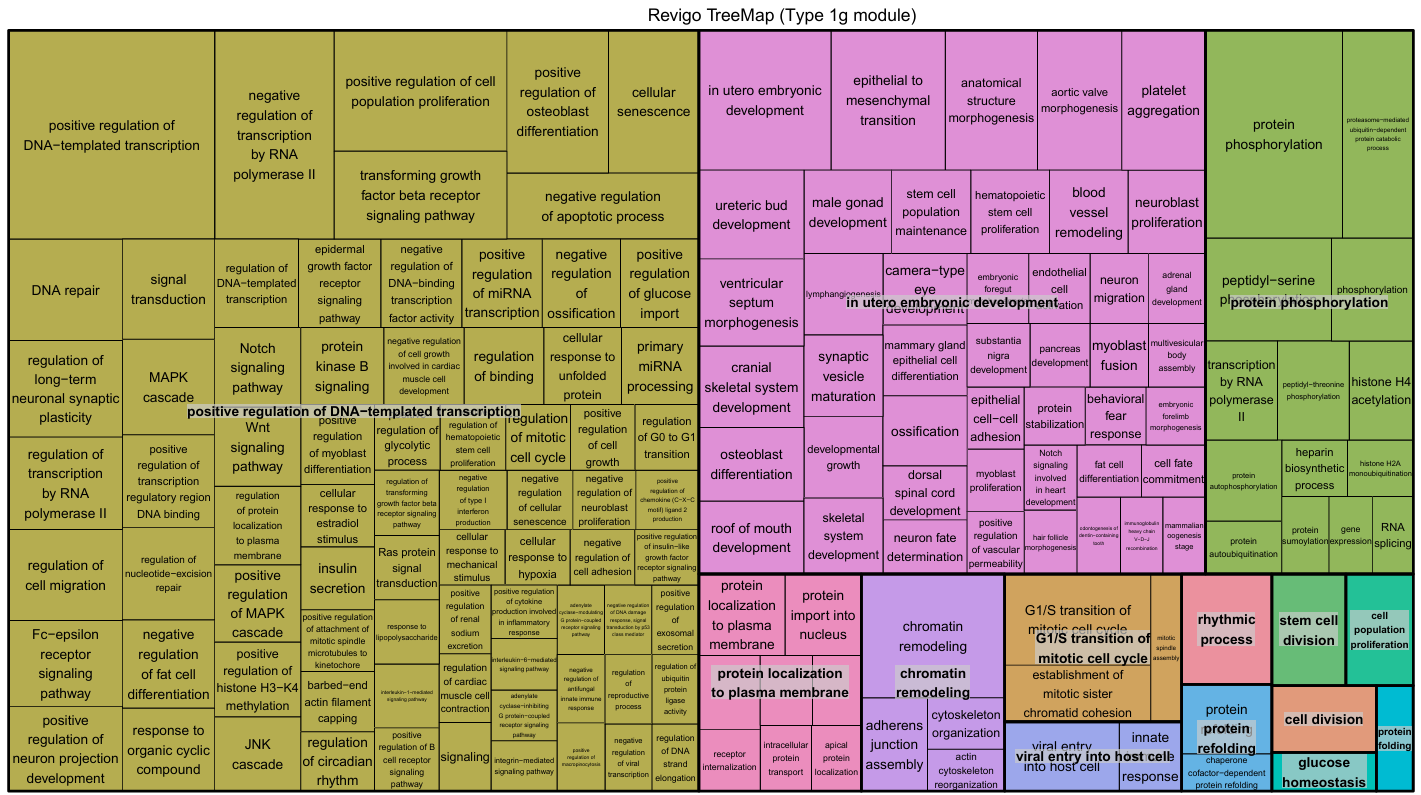


**Supplementary fig 15. Revigo treemap of significant GO enrichment terms for the Type 1g module.** The significant GO enrichment terms with *P* value ≤ 0.05 were identified by DAVID v6.8 pipeline.


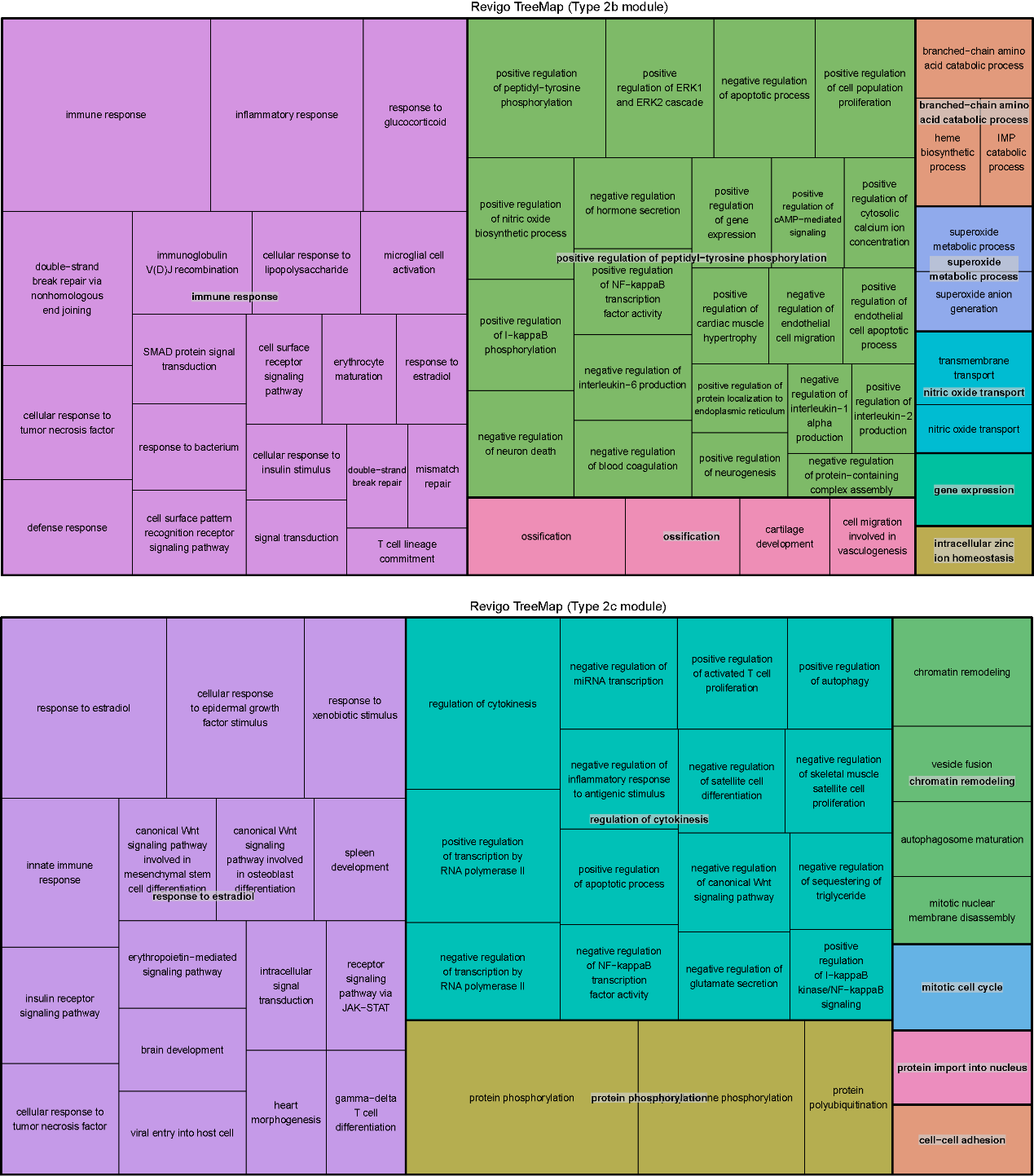


**Supplementary fig 16. Revigo treemap comparison of significant GO enrichment terms between the Type 2b and Type 2c modules.** The significant GO enrichment terms with *P* value ≤ 0.05 were identified by DAVID v6.8 pipeline.


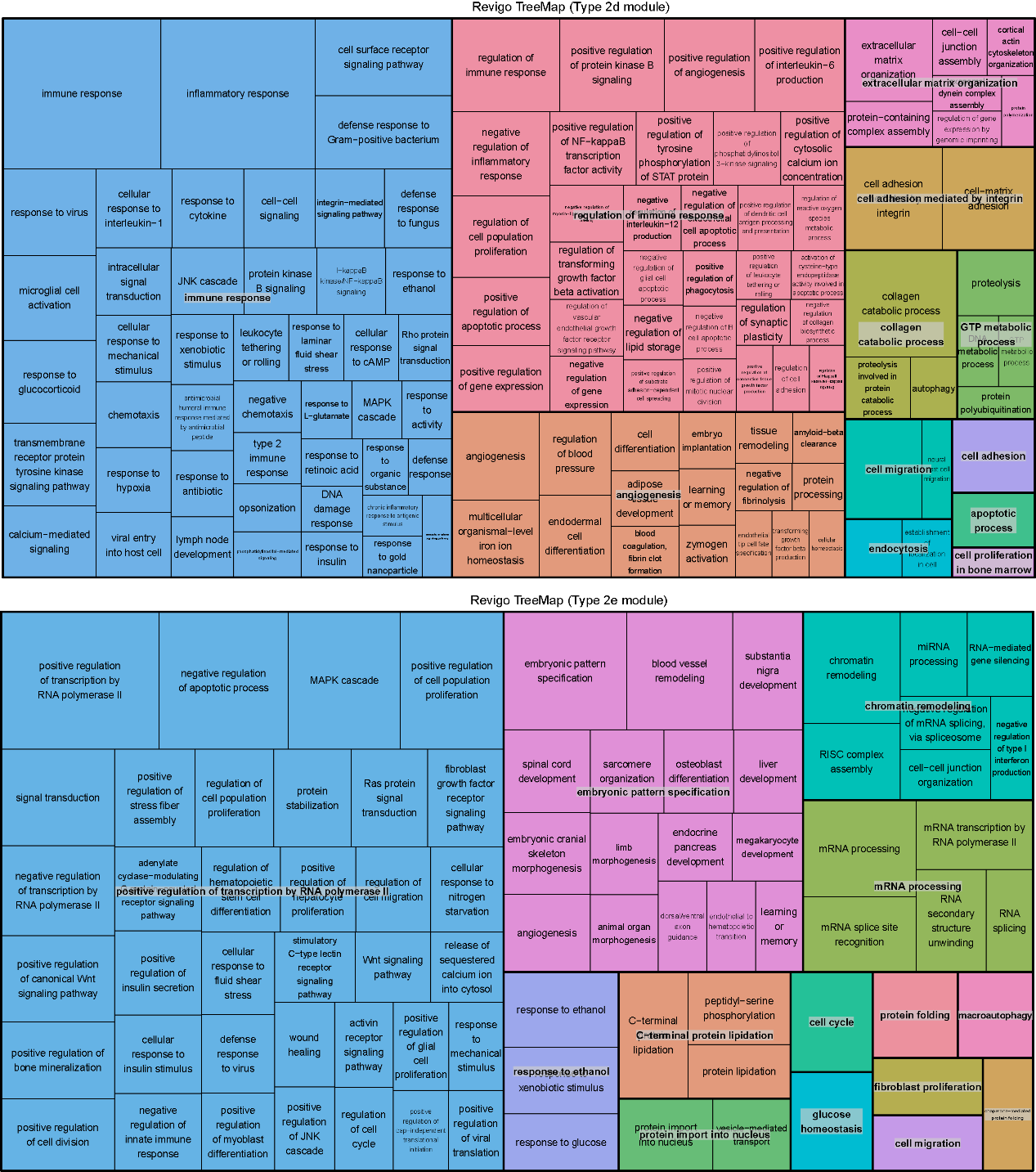


**Supplementary fig 17. Revigo treemap comparison of significant GO enrichment terms between the Type 2d and Type 2e modules.** The significant GO enrichment terms with *P* value ≤ 0.05 were identified by DAVID v6.8 pipeline.


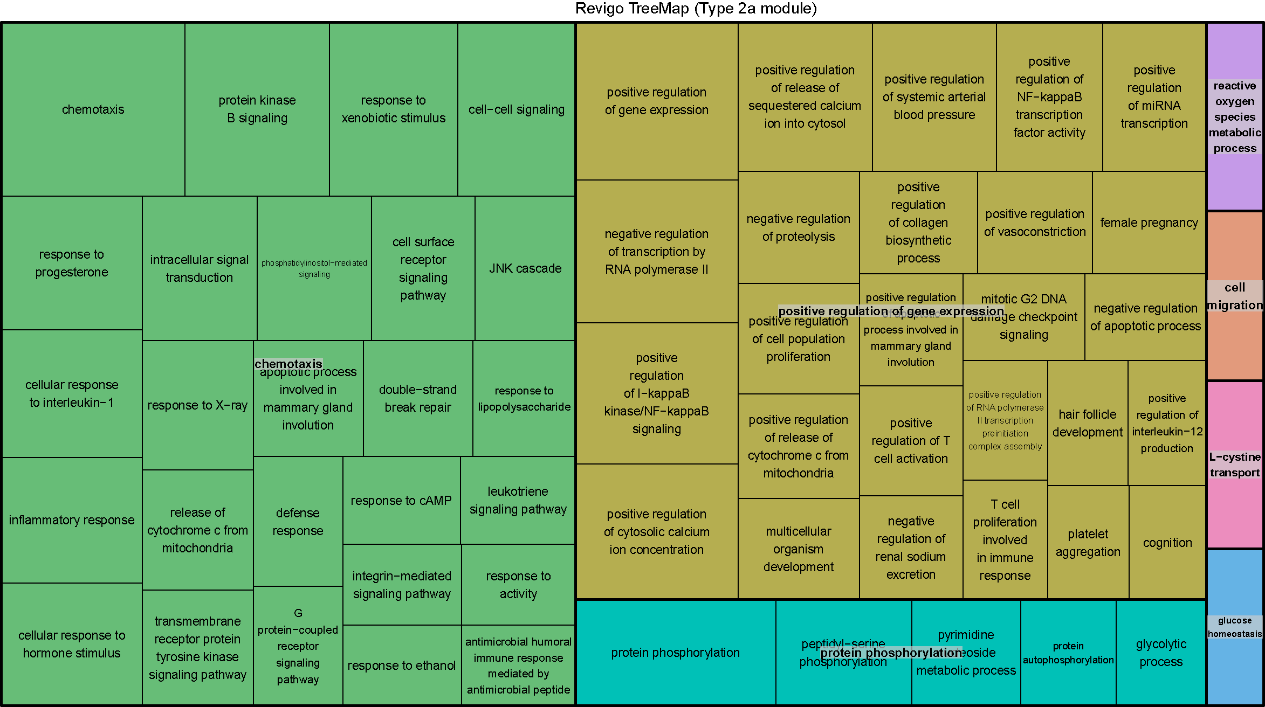


**Supplementary fig 18. Revigo treemap of significant GO enrichment terms for the Type 2a module.** The significant GO enrichment terms with *P* value ≤ 0.05 were identified by DAVID v6.8 pipeline.


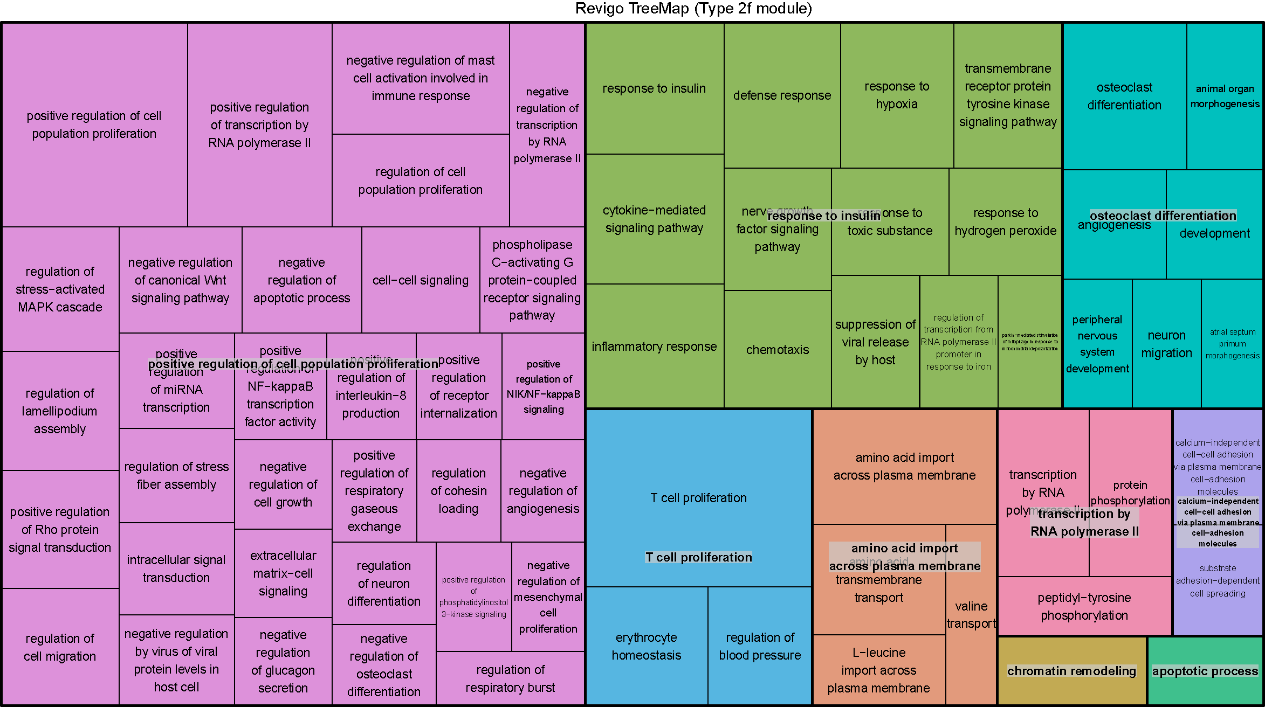


**Supplementary fig 19. Revigo treemap of significant GO enrichment terms for the Type 2f module.** The significant GO enrichment terms with *P* value ≤ 0.05 were identified by DAVID v6.8 pipeline.


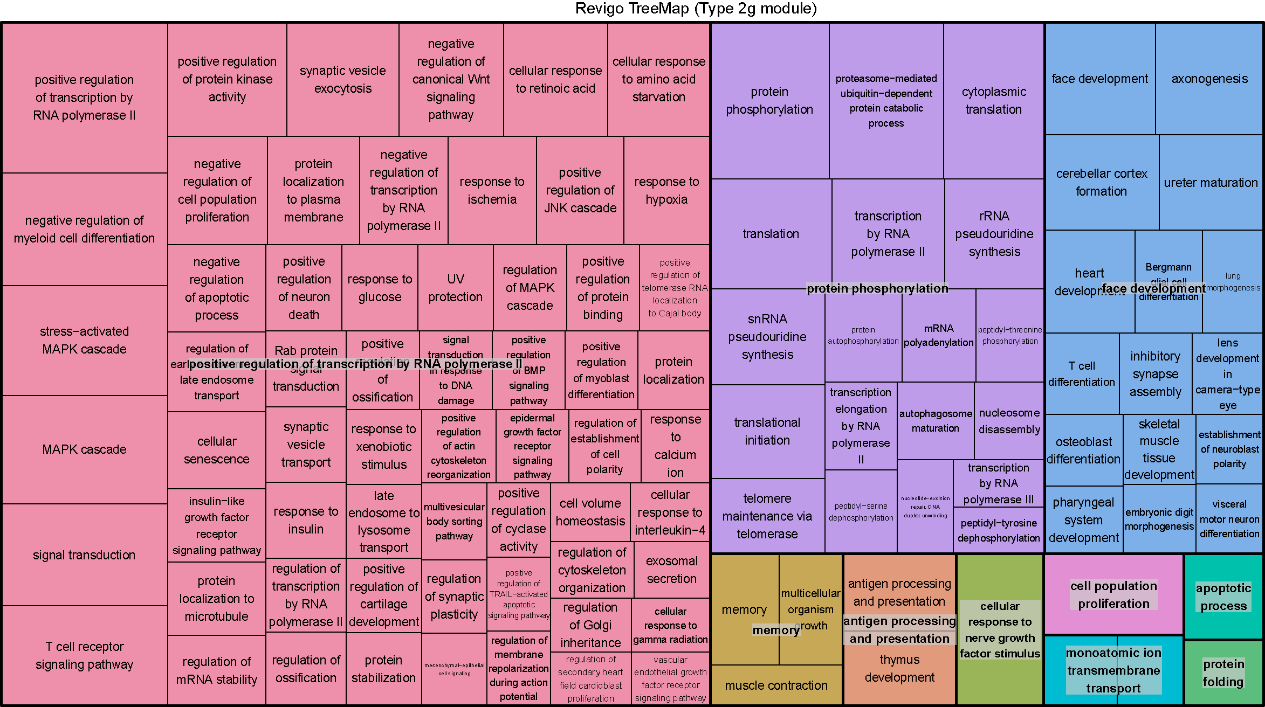


**Supplementary fig 20. Revigo treemap of significant GO enrichment terms for the Type 2g module.** The significant GO enrichment terms with *P* value ≤ 0.05 were identified by DAVID v6.8 pipeline.

**
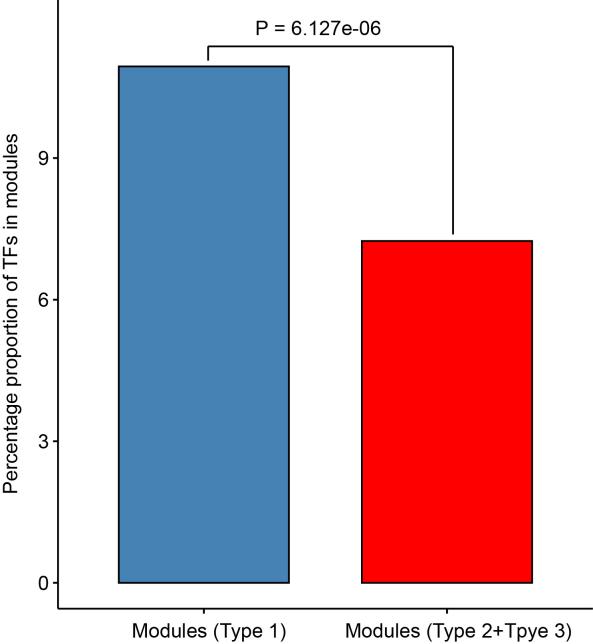
**

**Supplementary fig 21. Comparison of the percentage of transcription factors (TFs) present in Type 1 modules versus Type 2/3 modules.** Statistical significance was assessed using Fisher’s Exact Test.


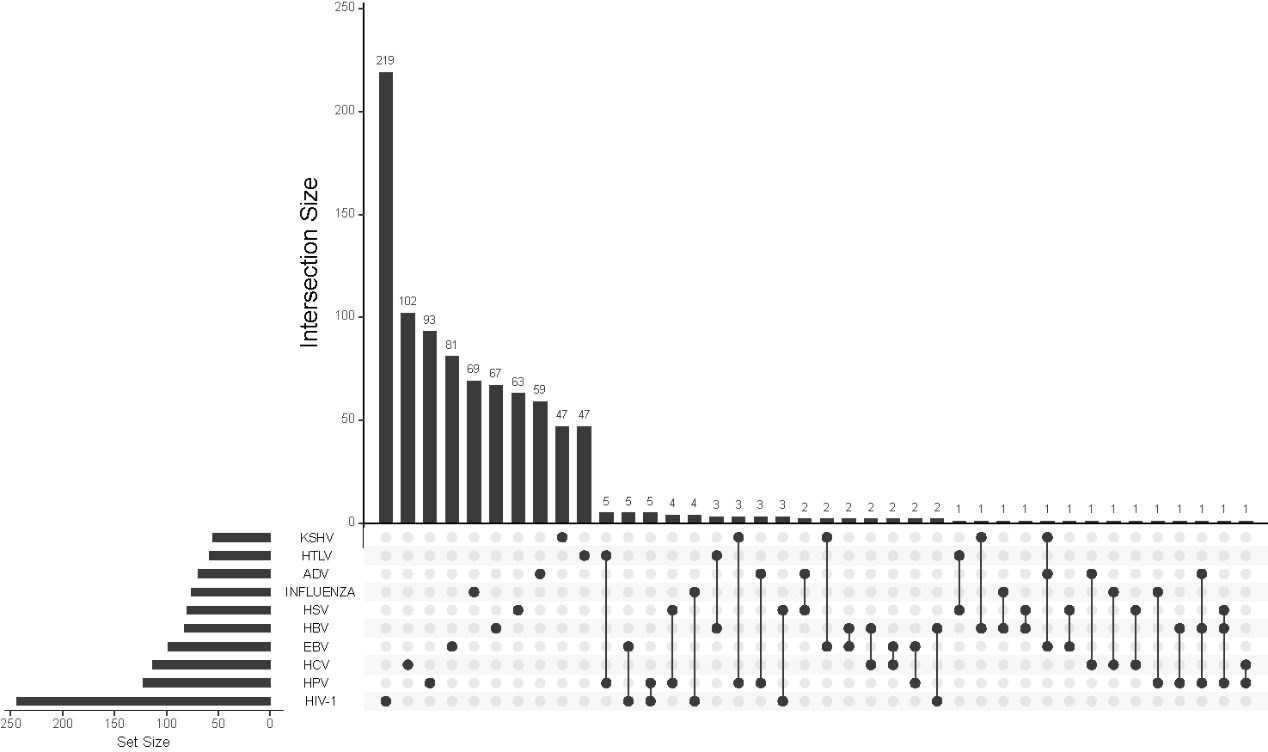


**Supplementary fig 22. Intersection size between the different sets for virus-interacting proteins.** These virus-interacting proteins were extracted from a previous study. HSV: herpes simplex virus. KSHV: kaposi’s sarcoma herpesvirus. HTLV: human T-lymphotropic virus. ADV: adenovirus. INFLUENZA: influenza virus. HBV: hepatitis B virus. HCV: hepatitis C virus. EBV: Epstein-barr virus. HPV: human papillomavirus. HIV-1: human immunodeficiency virus type 1.


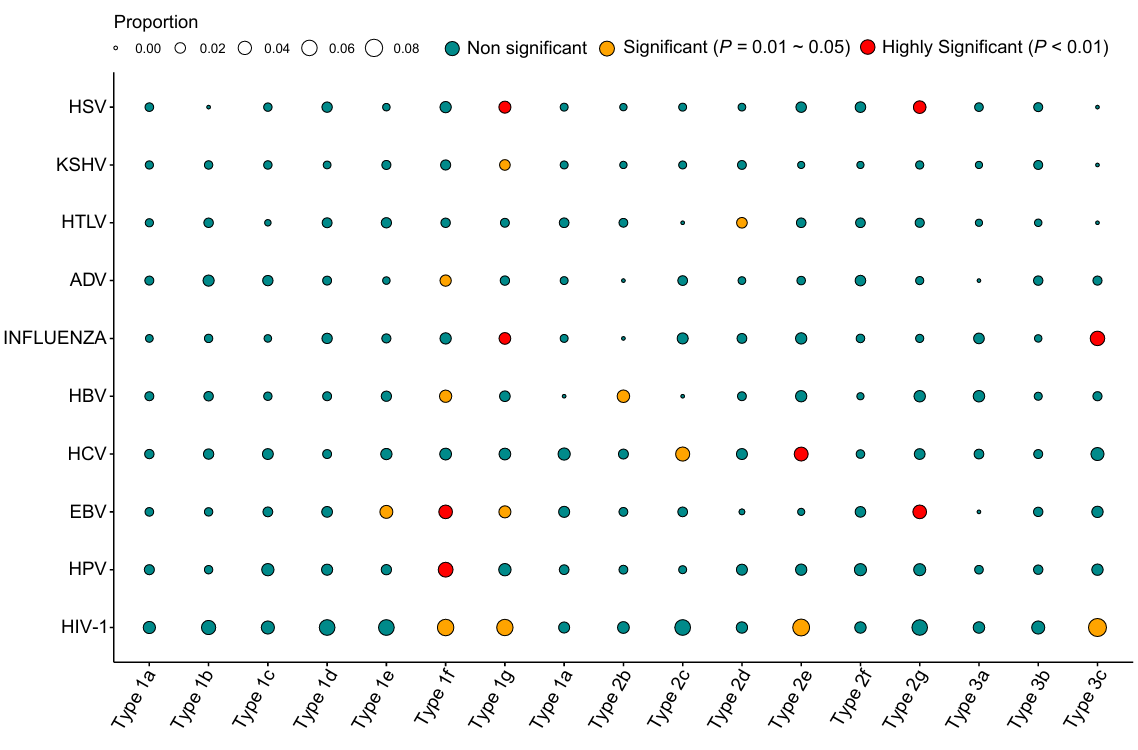


**Supplementary fig 23. Overlapping significant analyses between infection-related gene-sets and 17 modules by random sampling.** *P* values (≤ 0.05) of overlapping significance were decided by random sampling. HSV: herpes simplex virus. KSHV: kaposi’s sarcoma herpesvirus. HTLV: human T-lymphotropic virus. ADV: adenovirus. INFLUENZA: influenza virus. HBV: hepatitis B virus. HCV: hepatitis C virus. EBV: Epstein-barr virus. HPV: human papillomavirus. HIV-1: human immunodeficiency virus type 1. Note: For each gene module from this study, we generated 1,000 randomized gene sets from the full set of 4,714 immune-associated genes by randomly sampling with the identical number of module genes. Further, for each randomized gene set, we computed the number of overlapping genes between the randomized gene set and corresponding virus-interacting protein (VIP) list, to produce a null hypothesis distribution of expected overlaps. We then compared the observed overlap for each gene module with this null hypothesis distribution to assess whether the enrichment of VIPs in the module exceeded an expectation by chance with a higher proportion. The circle size represents the proportion of overlap among the module genes.


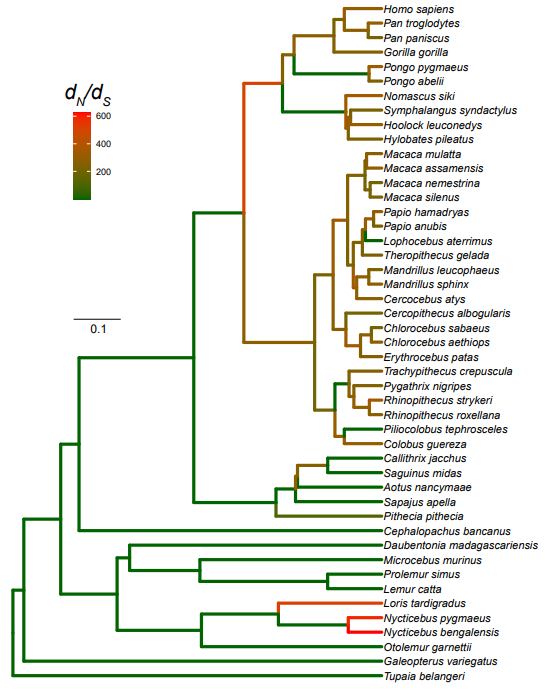


**Supplementary fig 24. The d*_N_/*d*_S_* values of the gene *EEF1G* in primate branches.** The d*_N_/*d*_S_* values were estimated using PAML4. The ruler represents the genetic distance of the primate phylogeny.


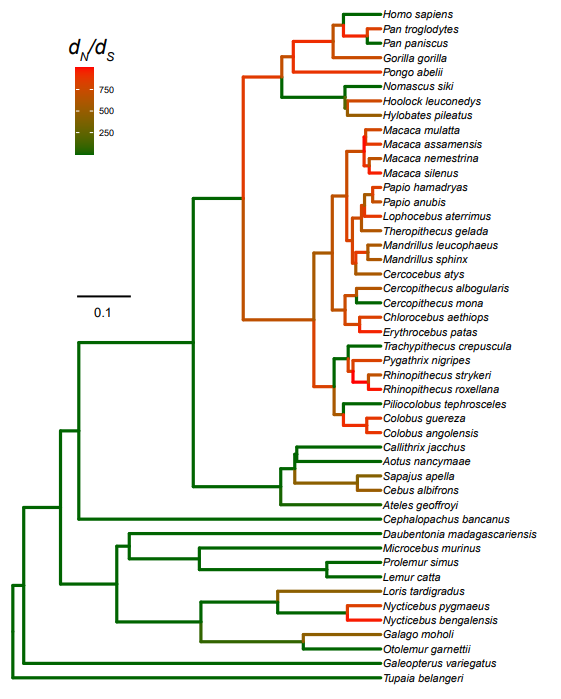
**Supplementary fig 25. The d*_N_/*d*_S_* values of the gene *EDC3* in primate branches.** The d*_N_/*d*_S_* values were estimated using PAML4. The ruler represents the genetic distance of the primate phylogeny.


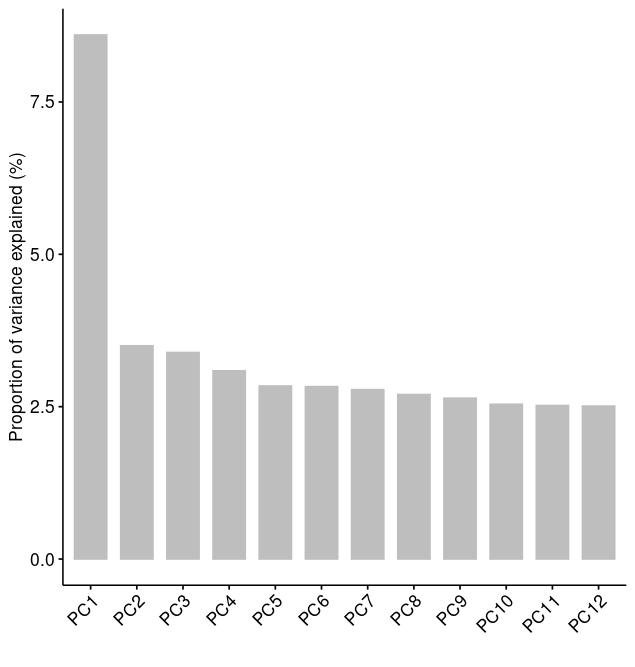


**Supplementary fig 26. Visualization of the variance explained by the first 12 PC axes.** The figure shows the percentage of variance explained for the top principal components based on d*_N_/*d*_S_* values across species.


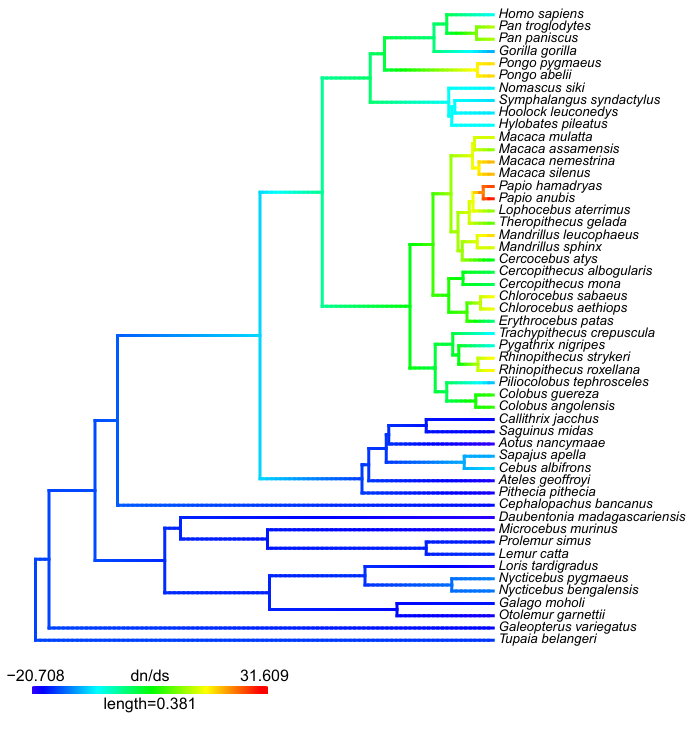


**Supplementary fig 27. Visualization of PC1 scores estimated by the d*_N_/*d*_S_* estimates using the primate phylogeny and the maximum likelihood reconstruction of the PC1 values for internal branches.** The PC1 scores explain 8.6% of the variance across all genes tested.


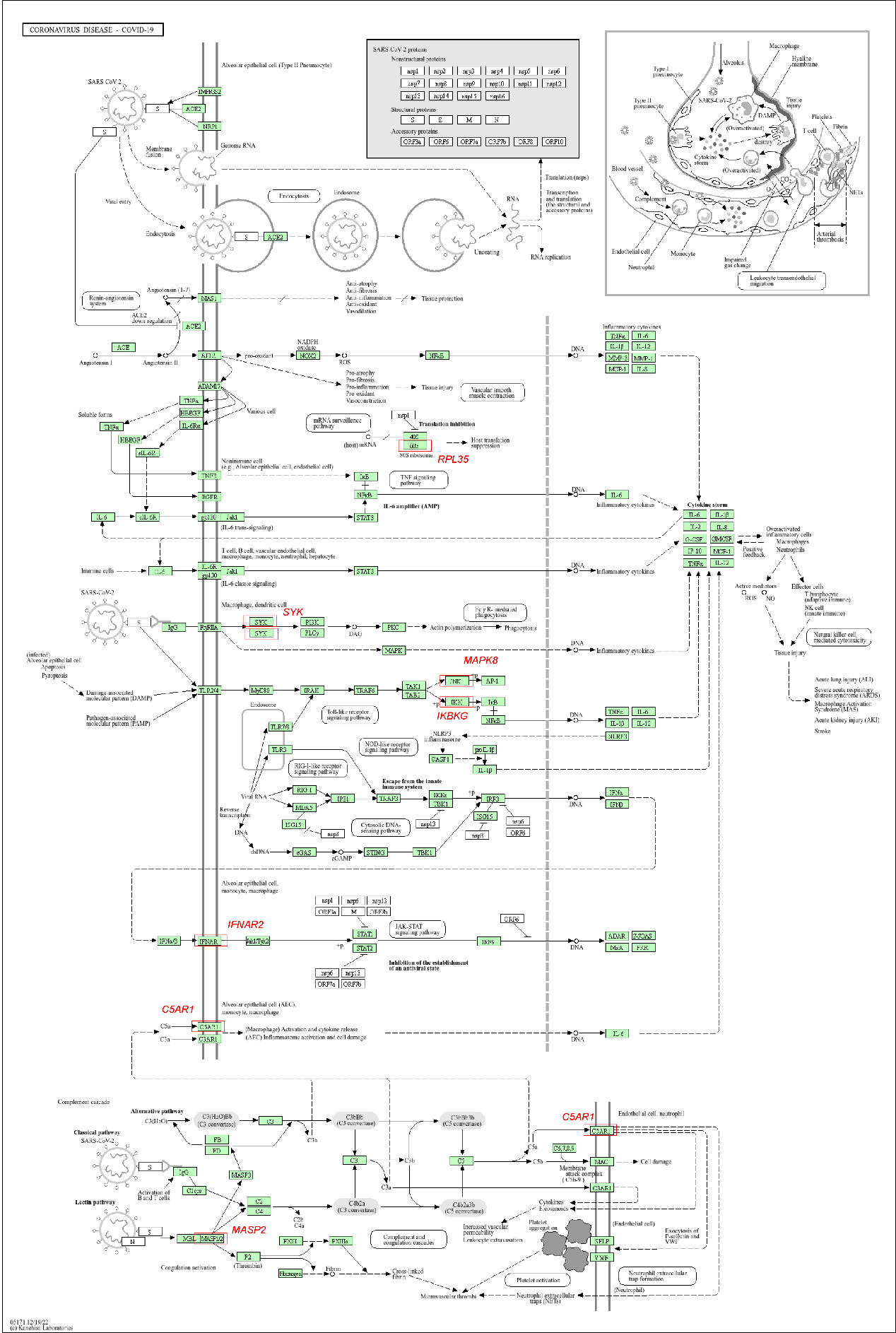


**Supplementary fig 28. Enrichment analyses of immune-associated genes (screened by social structure) involved in Coronavirus disease-COVID-19 signaling pathway.** Proteins associated with social structure were marked with the red boxes. Gene names were shown beside the protein names.

**Supplementary table 2. Genes having no variation in d*_N_*/d*_S_* across all branches of the 17 primate branches.** Note: for each gene, the d*_N_*/d*_S_* value was constant across the all 17 detected branches in this study.

| Ensembl Gene ID | *Gene Name* | d*_N_*/d*_S_* | d*_N_* in Primatomorpha; Primates; Strepsirrhini; Haplorrhini; Tarsiiformes; Simiiformes; Platyrrhini; Catarrhini; Cercopithecoidea; Hominoidea; Hylobatidae; Hominidae; *Pongo*; Homininae; Hominini; *Pan*; *Homo* |
| --- | --- | --- | --- |
| ENSG00000166407 | *LMO1* | 1.00E-04 | 3.3e-05;0;2e-06;4e-06;2e-05;7e-06;1e-06;3e-06;8e-06;1e-06;2e-06;0;2e-06;1e-06;0;0;0 |
| ENSG00000170027 | *YWHAG* | 1.00E-04 | 0.000118;8e-06;3e-06;4e-06;8e-06;4.2e-05;9e-06;1e-06;3e-06;2e-06;4e-06;2e-06;2e-06;3e-06;0;0;2e-06 |
| ENSG00000171148 | *TADA3* | 1.00E-04 | 7e-06;0;3e-06;3e-06;1.6e-05;0;3e-06;0;1.2e-05;0;9e-06;0;9e-06;0;0;0;0 |
| ENSG00000171720 | *HDAC3* | 1.00E-04 | 1e-06;1e-06;0;1e-06;1.4e-05;3e-06;1e-06;2e-06;2e-06;2e-06;0;0;1e-06;0;0;0;1e-06 |
| ENSG00000172819 | *RARG* | 1.00E-04 | 3e-06;0;3e-06;3e-06;1e-05;4e-06;3e-06;2e-06;2e-06;2e-06;1e-06;1e-06;2e-06;1e-06;0;0;1e-06 |
| ENSG00000173153 | *RSRRA* | 1.00E-04 | 3e-06;0;6e-06;9e-06;3.3e-05;1e-06;2e-06;2e-06;4e-06;1e-06;2e-06;1e-06;1e-06;1e-06;0;1e-06;0 |
| ENSG00000182944 | *EWSR1* | 1.00E-04 | 0;0;3e-05;0;0.000694;0;0;0;0;0;0;0;0;0;0;0;0 |
| ENSG00000183662 | *TAFA1* | 1.00E-04 | 0;0;5e-06;2e-06;4e-06;7e-06;2e-06;0;0;0;0;0;0;0;0;0;0 |
| ENSG00000184009 | *ACTG1* | 1.00E-04 | 5.4e-05;1.4e-05;0;5e-06;2.5e-05;1.7e-05;1.8e-05;1e-05;9e-06;9e-06;1e-05;4e-06;8e-06;5e-06;0;2e-06;3e-06 |
| ENSG00000197329 | *PELI1* | 1.00E-04 | 1e-06;2e-06;2e-06;0;8e-06;2e-06;1e-06;2e-06;2e-06;1e-06;1e-06;0;1e-06;1e-06;0;0;2e-06 |
| ENSG00000198648 | *STK39* | 1.00E-04 | 3e-06;1e-06;3e-06;0;8e-06;2e-06;1e-06;1e-06;2e-06;0;1e-06;0;1e-06;0;0;1e-06;0 |
| ENSG00000239306 | *RBM14* | 1.00E-04 | 4e-06;2e-06;1e-06;2e-06;2e-06;2e-06;4e-06;0;0;0;1e-06;0;2e-06;0;0;0;0 |
| ENSG00000241553 | *ARPC4* | 1.00E-04 | 0;0;5e-06;1e-06;1.2e-05;7e-06;5e-06;2e-06;1e-06;1e-06;0;1e-06;1e-06;0;0;0;1e-06 |
| ENSG00000100129 | *EIF3L* | 1.00E-04 | 4e-06;6e-06;2e-06;0;1.2e-05;6e-06;3e-06;2e-06;1e-06;1e-06;1e-06;0;3e-06;1e-06;0;2e-06;0 |
| ENSG00000100387 | *RBX1* | 1.00E-04 | 0;2e-06;1.4e-05;2e-06;4e-06;0;4e-06;2e-06;2e-06;4e-06;0;0;2e-06;0;0;0;0 |
| ENSG00000109670 | *FBXW7* | 1.00E-04 | 2e-06;1e-06;2e-06;1e-06;9e-06;2e-06;1e-06;0;2e-06;0;1e-06;0;0;0;0;0;0 |
| ENSG00000111875 | *ASF1A* | 1.00E-04 | 0;1e-06;5e-06;0;1.6e-05;4e-06;2e-06;1e-06;1e-06;0;0;0;0;0;0;1e-06;2e-06 |
| ENSG00000115524 | *SF3B1* | 1.00E-04 | 2e-06;1e-06;2e-06;0;1.7e-05;5e-06;3e-06;1e-06;2e-06;0;1e-06;0;1e-06;1e-06;0;0;1e-06 |
| ENSG00000115966 | *ATF2* | 1.00E-04 | 0;1e-06;1e-06;0;7e-06;1e-06;1e-06;0;1e-06;1e-06;0;0;1e-06;1e-06;0;0;0 |
| ENSG00000125995 | *ROMO1* | 1.00E-04 | 0;0;9e-06;0;1.1e-05;4e-06;4e-06;2e-06;0;0;0;0;0;2e-06;0;2e-06;2e-06 |
| ENSG00000132017 | *DCAF15* | 1.00E-04 | 2e-06;9e-06;1.5e-05;0;3e-05;3.2e-05;2.1e-05;4e-06;7e-06;5e-06;7e-06;1e-06;2e-06;7e-06;0;0;2e-06 |
| ENSG00000133703 | *KRAS* | 1.00E-04 | 1e-06;1e-06;1e-06;0;3e-06;1e-06;1e-06;0;0;1e-06;0;0;0;0;0;0;0 |
| ENSG00000135414 | *GDF11* | 1.00E-04 | 1e-06;3e-06;5e-06;1e-06;1.6e-05;5e-06;3e-06;2e-06;1e-06;0;0;1e-06;2e-06;0;0;0;0 |
| ENSG00000137168 | *PPIL1* | 1.00E-04 | 5e-05;5e-06;2e-06;0;7e-06;6e-06;2e-06;1e-06;1e-06;0;1e-06;0;2e-06;3e-06;0;0;0 |
| ENSG00000055130 | *CUL1* | 1.00E-04 | 1e-06;4e-06;6e-06;1e-06;1.6e-05;5e-06;4e-06;3e-06;2e-06;1e-06;1e-06;0;1e-06;0;0;1e-06;0 |
| ENSG00000138663 | *COPS4* | 1.00E-04 | 2e-06;1e-06;2e-06;0;1.5e-05;3e-06;3e-06;2e-06;1e-06;1e-06;1e-06;0;1e-06;0;0;0;0 |
| ENSG00000058262 | *SEC61A1* | 1.00E-04 | 2.1e-05;5e-06;5e-06;0;5e-06;0;5e-06;5e-06;0;0;0;0;0;0;0;0;0 |
| ENSG00000143632 | *ACAT1* | 1.00E-04 | 6.4e-05;1.5e-05;2e-06;7e-06;9.2e-05;1.7e-05;7e-06;7e-06;1.1e-05;2e-06;3e-06;2e-06;1e-06;2e-06;0;2e-06;1e-06 |
| ENSG00000143995 | *MEIS1* | 1.00E-04 | 0;0;1e-06;0;5e-06;1e-06;1e-06;1e-06;1e-06;0;1e-06;0;1e-06;1e-06;0;0;0 |
| ENSG00000149923 | *PPP4C* | 1.00E-04 | 0;0;3e-06;1e-06;8e-06;1.5e-05;3e-06;4e-06;7e-06;0;1e-06;2e-06;1e-06;2e-06;1e-06;1e-06;1e-06 |

**Supplementary table 4. Enrichment analysis of TFs in 17 gene modules.** The *P*-values were decided by Fisher Exact Test. TFs: transcription factors.

| Cluster | *P*-value | Odds ratio |
| --- | --- | --- |
| Type 1f | 6.67E-06 | 2.199163146 |
| Type 1g | 0.000602926 | 1.675371963 |
| Type 1e | 0.000627646 | 2.150789325 |
| Type 2g | 0.05603329 | 1.42759936 |
| Type 2a | 0.082011849 | 1.515160597 |
| Type 2f | 0.129885785 | 1.331141198 |
| Type 2c | 0.205720704 | 1.306770806 |
| Type 1b | 0.219743035 | 1.221050469 |
| Type 2e | 0.298143121 | 1.160738503 |
| Type 1a | 0.812660427 | 0.878010987 |
| Type 1d | 0.827313115 | 0.852508167 |
| Type 2b | 0.909346524 | 0.701546589 |
| Type 1c | 0.910814623 | 0.762120749 |
| Type 3c | 0.943298511 | 0.518606554 |
| Type 2d | 0.999469528 | 0.524081776 |
| Type 3a | 0.999948687 | 0.255700855 |
| Type 3b | 0.999999974 | 0.318644367 |

**Supplementary table 5. Genes having no variation in d*_N_*/d*_S_* across 50 terminal branches of the primate phylogeny.** Note: for each gene, the d*_N_*/d*_S_* value was constant across the all 50 detected branches in this study. The d*_N_* and d*_S_* values were exhibited here across each of all terminal branches. NA represented the missing value.

| **Ensembl Gene ID** | ***Gene***  ***Name*** | **d*_N_*/d*_S_*** | **(d*_N_,* d*_S_*) in *Homo sapiens; Pan troglodytes; Pan paniscus; Gorilla gorilla; Pongo pygmaeus; Pongo abelii; Nomascus siki; Symphalangus syndactylus; Hoolock leuconedys; Hylobates pileatus; Macaca mulatta; Macaca assamensis; Macaca nemestrina; Macaca Silenus; Papio hamadryas; Papio Anubis; Lophocebus aterrimus; Theropithecus gelada; Mandrillus leucophaeus; Mandrillus sphinx; Cercocebus atys; Cercopithecus albogularis; Cercopithecus mona; Chlorocebus aethiops; Chlorocebus sabaeus; Erythrocebus patas; Trachypithecus crepuscula; Pygathrix nigripes; Rhinopithecus strykeri; Rhinopithecus roxellana; Piliocolobus tephrosceles; Colobus guereza; Colobus angolensis; Callithrix jacchus; Saguinus midas; Aotus nancymaae; Sapajus apella; Cebus albifrons; Ateles geoffroyi; Pithecia pithecia; Cephalopachus bancanus; Microcebus murinus; Prolemur simus; Lemur catta; Daubentonia madagascariensis; Loris tardigradus; Nycticebus pygmaeus; Nycticebus bengalensis; Galago moholi; Otolemur garnettii*** |
| --- | --- | --- | --- |
| ENSG00000058262 | *SEC61A1* | 1.00E-04 | (0, 5.00E-06); (0, 5.00E-06); (0, 5.00E-06); (0, 5.00E-06); (0, 5.00E-06); (0, 5.00E-06); (5.00E-06, 0.054331); (0, 5.00E-06); (0, 5.00E-06); (0, 5.00E-06); (0, 5.00E-06); (0, 5.00E-06); (0, 5.00E-06); (0, 5.00E-06); (0, 5.00E-06); (0, 5.00E-06); (0, 5.00E-06); (0, 5.00E-06); (0, 5.00E-06); (0, 5.00E-06); (0, 5.00E-06); (0, 5.00E-06); (0, 5.00E-06); (0, 5.00E-06); (0, 5.00E-06); (0, 5.00E-06); (0, 5.00E-06); (5.00E-06, 0.053177); (0, 5.00E-06); (0, 5.00E-06); (0, 5.00E-06); (0, 5.00E-06); (0, 5.00E-06); (0, 5.00E-06); (0, 5.00E-06); (0, 5.00E-06); (0, 5.00E-06); (NA, NA;) (5.00E-06, 0.052321); (0, 5.00E-06); (5.00E-06, 0.052251); (0.001074, 10.74061); (NA, NA;) (3.90E-05, 0.386022); (0, 5.00E-06); (0, 5.00E-06); (0, 5.00E-06); (5.00E-06, 0.053393); (5.00E-06, 0.053393); (0, 5.00E-06) |
| ENSG00000077080 | *ACTL6B* | 1.00E-04 | (2.00E-06, 0.016008); (0, 0.00393); (0, 6.00E-06); (NA, NA;) (0, 0.003956); (1.00E-06, 0.00781); (1.00E-06, 0.011803); (1.00E-06, 0.011811); (0, 6.00E-06); (1.00E-06, 0.011815); (1.00E-06, 0.008015); (0, 6.00E-06); (0, 6.00E-06); (1.00E-06, 0.008003); (0, 6.00E-06); (0, 0.004003); (0, 6.00E-06); (0, 0.004031); (0, 0.004032); (0, 0.004035); (NA, NA;) (0, 0.004105); (2.00E-06, 0.024273); (1.00E-06, 0.008059); (0, 6.00E-06); (NA, NA;) (NA, NA;) (1.00E-06, 0.008137); (1.00E-06, 0.008009); (NA, NA;) (2.00E-06, 0.016292); (0, 6.00E-06); (0, 0.003981); (7.00E-06, 0.066996); (NA, NA;) (5.00E-06, 0.051255); (4.00E-06, 0.038627); (1.00E-06, 0.005159); (6.00E-06, 0.059459); (6.00E-06, 0.057553); (3.50E-05, 0.350374); (7.00E-06, 0.065285); (1.00E-06, 0.012742); (2.00E-06, 0.02487); (6.00E-06, 0.063407); (5.00E-06, 0.048085); (1.00E-06, 0.00756); (1.00E-06, 0.010822); (NA, NA;) (5.00E-06, 0.053615) |
| ENSG00000100387 | *RBX1* | 1.00E-04 | (0, 5.00E-06); (2.00E-06, 0.01785); (0, 5.00E-06); (2.00E-06, 0.017838); (0, 5.00E-06); (0, 5.00E-06); (0, 5.00E-06); (NA, NA;) (0, 5.00E-06); (0, 5.00E-06); (0, 5.00E-06); (0, 5.00E-06); (0, 5.00E-06); (0, 5.00E-06); (0, 5.00E-06); (0, 5.00E-06); (0, 5.00E-06); (0, 5.00E-06); (0, 5.00E-06); (0, 5.00E-06); (0, 5.00E-06); (0, 5.00E-06); (2.00E-06, 0.017823); (0, 5.00E-06); (0, 5.00E-06); (NA, NA;) (NA, NA;) (0, 5.00E-06); (0, 5.00E-06); (0, 5.00E-06); (NA, NA;) (7.00E-06, 0.073604); (0, 5.00E-06); (0, 5.00E-06); (2.00E-06, 0.017843); (0, 5.00E-06); (2.00E-06, 0.017858); (0, 5.00E-06); (0, 5.00E-06); (0, 5.00E-06); (4.00E-06, 0.036526); (4.00E-06, 0.035999); (4.00E-06, 0.037599); (2.00E-06, 0.018138); (2.00E-06, 0.019236); (2.00E-06, 0.017935); (0, 5.00E-06); (0, 5.00E-06); (0, 5.00E-06); (0, 5.00E-06) |
| ENSG00000144566 | *RAB5A* | 1.00E-04 | (0, 3.00E-06); (0, 3.00E-06); (NA, NA;) (0, 3.00E-06); (NA, NA;) (0, 3.00E-06); (0, 3.00E-06); (0, 3.00E-06); (NA, NA;) (NA, NA;) (NA, NA;) (0, 3.00E-06); (NA, NA;) (NA, NA;) (0, 3.00E-06); (0, 3.00E-06); (NA, NA;) (0, 3.00E-06); (0, 3.00E-06); (NA, NA;) (0, 3.00E-06); (0, 3.00E-06); (NA, NA;) (0, 3.00E-06); (NA, NA;) (NA, NA;) (0.000345, 3.452763); (NA, NA;) (0, 3.00E-06); (0, 3.00E-06); (NA, NA;) (0, 3.00E-06); (0, 3.00E-06); (0, 3.00E-06); (0, 3.00E-06); (0, 3.00E-06); (0, 3.00E-06); (NA, NA;) (0, 3.00E-06); (NA, NA;) (6.40E-05, 0.642529); (0, 3.00E-06); (0, 3.00E-06); (0, 3.00E-06); (0, 3.00E-06); (0, 3.00E-06); (0, 3.00E-06); (0, 3.00E-06); (NA, NA;) (0, 3.00E-06) |

**Supplementary table 8. Results for residual testis mass, social structure, group size and diet models: PC2 ~ residual testis mass + Social structure + group size (log)+diet.**

| **Branch length comparisons** | **dBIC** | **Weight** |
| --- | --- | --- |
| **Kappa** | **0.0** | **0.996** |
| Delta | 12.16 | 0.002 |
| Lambda | 13.02 | 0.001 |
| **Sequential SS ANOVA** | **Mean sq.** | ***P* value** |
| **Residual testis mass** | **102.225** | **0.03469** |
| Social structure | 48.406 | 0.09558 |
| Group size (log) | 3.523 | 0.68455 |
| Diet | 19.921 | 0.42806 |
| Residuals | 20.947 |  |

**Supplementary table 9. Results for residual testis mass, social structure, group size and diet models: PC3 ~ residual testis mass + Social structure + group size (log)+diet.**

| **Branch length comparisons** | **dBIC** | **Weight** |
| --- | --- | --- |
| **Lambda** | **0.0** | **0.611** |
| Kappa | 0.9652 | 0.377 |
| Delta | 7.7800 | 0.012 |
| **Sequential SS ANOVA** | **Mean sq.** | ***P* value** |
| Residual testis mass | 308.36 | 0.14161 |
| **Social structure** | **451.17** | **0.03221** |
| Group size (log) | 100.45 | 0.39594 |
| Diet | 114.04 | 0.48169 |
| Residuals | 135.55 |  |

**Supplementary table 10. Results for residual testis mass, social structure, group size and diet models: PC4 ~ residual testis mass + Social structure + group size (log)+diet.**

| **Branch length comparisons** | **dBIC** | **Weight** |
| --- | --- | --- |
| **Lambda** | **0.000** | **0.697** |
| Kappa | 1.672 | 0.302 |
| Delta | 12.300 | 0.001 |
| **Sequential SS ANOVA** | **Mean sq.** | ***P* value** |
| Residual testis mass | 317.00 | 0.1416 |
| Social structure | 257.95 | 0.1585 |
| Group size (log) | 129.17 | 0.3431 |
| Diet | 54.04 | 0.7625 |
| Residuals | 139.35 |  |
